# DNA aptamers against bacterial cells can be efficiently selected by a SELEX process using state-of-the art qPCR and ultra-deep sequencing

**DOI:** 10.1038/s41598-020-77221-9

**Published:** 2020-12-01

**Authors:** Claudia Kolm, Isabella Cervenka, Ulrich J. Aschl, Niklas Baumann, Stefan Jakwerth, Rudolf Krska, Robert L. Mach, Regina Sommer, Maria C. DeRosa, Alexander K. T. Kirschner, Andreas H. Farnleitner, Georg H. Reischer

**Affiliations:** 1 Molecular Diagnostics Group, Department IFA-Tulln, Institute of Chemical, Environmental and Bioscience Engineering, TU Wien, Tulln, Austria; 2 ICC Interuniversity Cooperation Centre Water and Health, Vienna, Austria; 3 Institute for Hygiene and Applied Immunology, Medical University Vienna, Vienna, Austria; 4 Institute of Bioanalytics and Agro-Metabolomics, Department IFA-Tulln, University of Natural Resources and Life Sciences, Vienna (BOKU), Tulln, Austria; 5 School of Biological Sciences, Institute for Global Food Security, Queen’s University Belfast, Belfast, Northern Ireland, UK; 6 Research Group Synthetic Biology and Molecular Biotechnology (166-5-1), Institute of Chemical, Environmental and Bioscience Engineering, TU Wien, Vienna, Austria; 7 Department of Chemistry, Carleton University, Ottawa, Canada; 8 Research Unit Water Quality and Health, Department Physiology, Pharmacology and Microbiology, Karl Landsteiner University of Health Sciences, Krems, Austria; 9 Research Group Environmental Microbiology and Molecular Diagnostics (166-5-3), Institute of Chemical, Environmental and Bioscience Engineering, TU Wien, Vienna, Austria

**Keywords:** DNA, Bacteria

## Abstract

DNA aptamers generated by cell-SELEX against bacterial cells have gained increased interest as novel and cost-effective affinity reagents for cell labelling, imaging and biosensing. Here we describe the selection and identification of DNA aptamers for bacterial cells using a combined approach based on cell-SELEX, state-of-the-art applications of quantitative real-time PCR (qPCR), next-generation sequencing (NGS) and bioinformatic data analysis. This approach is demonstrated on *Enterococcus faecalis* (*E. faecalis*), which served as target in eleven rounds of cell-SELEX with multiple subtractive counter-selections against non-target species. During the selection, we applied qPCR-based analyses to evaluate the ssDNA pool size and remelting curve analysis of qPCR amplicons to monitor changes in pool diversity and sequence enrichment. Based on NGS-derived data, we identified 16 aptamer candidates. Among these, aptamer EF508 exhibited high binding affinity to *E. faecalis* cells (K_D_-value: 37 nM) and successfully discriminated *E. faecalis* from 20 different *Enterococcus* and non-*Enterococcus* spp. Our results demonstrate that this combined approach enabled the rapid and efficient identification of an aptamer with both high affinity and high specificity. Furthermore, the applied monitoring and assessment techniques provide insight into the selection process and can be highly useful to study and improve experimental cell-SELEX designs to increase selection efficiency.

## Introduction

DNA aptamers are short synthetic oligonucleotides (20–100 nucleotides) that can bind to a molecular target by their unique three-dimensional structure with high affinity and specificity. In contrast to their antibody-counterparts, aptamers offer many inherent advantages, which make them appealing alternatives in diverse analytical applications. First, aptamers can be readily synthesized in large quantities at relatively low costs and with low batch-to-batch variations. Due to chemical synthesis, they can be easily modified with chemical groups and fluorescence dyes in a targeted manner for immobilization and signalling purposes. Second, they are relatively small in terms of their molecular weight (10–30 kDa), which allows them to reach epitopes that antibodies (~ 150 kDa) cannot target due to steric hindrance^1^. Third, aptamers offer higher stability and can be regenerated by de/renaturation without losing their functionality, which is extremely useful, especially for biosensor development. However, the most appealing feature of aptamers is that they can be selected in vitro through an iterative process termed SELEX (Systematic Evolution of Ligands by EXponential enrichment)^2,3^, which can be tailored to the needs of the intended application. This process has been used over the past decades to select aptamers for a wide range of targets, from small molecules such as organic dyes^4^, cocaine^5^ or mycotoxins^6,7^ to numerous proteins such as thrombin^8^, tumour marker peptides^9^ and growth factors^10^ as well as complex targets such as mammalian cells^11^. More recently, bacterial cells have also become frequent targets in aptamer selection processes.

Currently, the discovery of aptamers for bacterial cells is mainly based on whole cell-SELEX, which is a modified form of the conventional SELEX procedure that uses purified target molecules^12^. In cell-SELEX, DNA aptamers typically evolve from large randomized single-stranded DNA (ssDNA) libraries (consisting of up to 10^15^ unique sequences) by repetitive binding to whole bacterial target cells and partitioning by centrifugation. Over the course of the selection process, cell-bound ssDNA fractions are repeatedly amplified by PCR and regenerated for the next round of cell-SELEX. After multiple rounds of selection (typically 6–20 rounds), the final ssDNA pool is traditionally cloned into plasmids followed by Sanger sequencing to identify aptamer candidates. In contrast to conventional SELEX using isolated cell-surface proteins that are immobilized on solid supports, whole cell-SELEX offers the inherent advantage that it allows selecting aptamers that interact directly with cellular epitopes in their native conformation with no prior knowledge of the molecular target^12^. In this way, aptamers have been selected for human and food-borne pathogens, such as *Streptococcus spp.*^13,14^, *Listeria monocytogenes*^15^, *Salmonella* spp.^16,17^, *Staphylococcus aureus*^18^, *Vibrio parahaemolyticus*^19^, *Escherichia coli*^20^ and *Pseudomonas aeruginosa*^21^.

Despite success in aptamer discovery, bacterial whole cell-SELEX suffers from methodological challenges. Many steps of the procedure are performed in a semi-blind fashion with limited control over and insight into how the ssDNA pool is evolving. This black box can make SELEX a time-consuming, labour-intensive and costly trial and error process. Also, the complex nature of bacterial cells with thousands of cell-surface molecules and structures leads to a much larger number of different aptamer sequences that are enriched in parallel compared to single and defined targets. Thus, traditional cloning and Sanger sequencing of 30–100 clones (e.g. Refs.^22–24^) from the last round of whole cell-SELEX can hinder the identification of aptamer candidates with desired binding characteristics. To address these limitations, next-generation sequencing (NGS) is increasingly replacing the low-throughput sequencing step in SELEX procedures^25–27^. NGS enables the analysis of millions of sequences and early identification of high-quality aptamers, which in turn can reduce the overall number of selection rounds required. Furthermore, complementary methods, such as quantitative real-time PCR (qPCR) have been proposed for SELEX monitoring and assessment on a round-to-round basis. For instance, qPCR was used to quantify the amount of bound ssDNA on human embryonic kidney cells^28^. In contrast to spectrophotometric measurements (e.g. Nanodrop) or fluorescence-based binding assays (e.g. flow cytometry), qPCR is highly sensitive and requires no labelling of ssDNA for the detection. Simultaneously, the quantification of cell-bound ssDNA enables the determination of the optimal (minimal) number of PCR cycles for amplifying the cell-bound fractions for the next round of SELEX^28^. This reduces the risk of PCR by-products and artefacts that often contaminate the enriched pool and render the selection ineffective^28,29^. Moreover, qPCR-based remelting curve analysis has been proposed to monitor changes in sequence diversity^30–32^. Based on the denaturation of double-stranded qPCR products after reannealing under stringent conditions, VanBrabant et al. applied remelting curve analysis to gain information on enrichment in steroid, peptide and protein targeting SELEX procedures, suggesting its general applicability as a monitoring tool^31^.

In the present study, we describe a combined approach that incorporates whole cell-SELEX, qPCR-based monitoring tools, NGS and custom-designed bioinformatic data analysis to select and identify DNA aptamers for *Enterococcus faecalis* (*E. faecalis*) cells. *E. faecalis* served primarily as a model organism, which we chose for the following reasons. *E. faecalis* is a commensal gut bacterium, which is widely used together with other enterococci as faecal indicator for assessing water quality^33^. Likewise, it is an opportunistic pathogen with increasing antibiotic resistance^34^. Based upon this health-relevant bacterium, we demonstrate the individual processes and the utility of this new and combined aptamer discovery approach for generating bacterial cell-recognizing aptamers.

## Results and discussion

### In vitro selection of aptamers specific for *E. faecalis*

**Figure 1:**
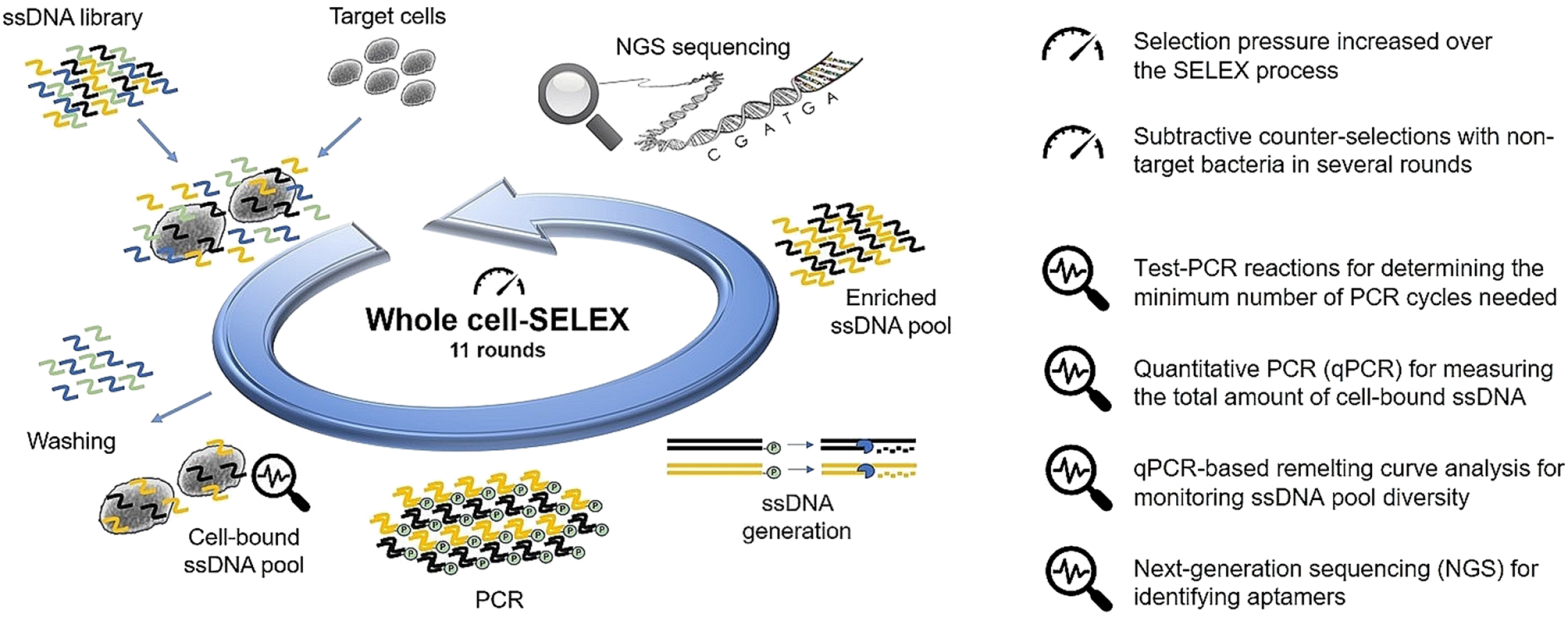
Schematic illustration of the bacterial whole cell-SELEX process in combination with qPCR-based and NGS tools. A total of eleven SELEX rounds of positive selection with *E. faecalis* cells as target were performed. In rounds R03, R04, R05, R06 and R09 subtractive counter-selections were carried out with mixtures of enterococcal and non-enterococcal species, respectively, which were incubated with the enriched ssDNA pool prior to positive selection.

A general outline of the combined aptamer discovery approach developed to select DNA aptamers for whole bacterial cells is depicted in Fig. 1. Here we used a customized cell-SELEX protocol^12^ and performed eleven rounds of SELEX with *E. faecalis* type strain DSM-20478 as target cells. Following incubation with the random ssDNA library, cells were washed by centrifugation to remove unbound sequences. Sequences bound to the cells were amplified by PCR and rendered single stranded by Lambda-exonuclease digestion for the next round of SELEX. In each round, samples of the cell-bound ssDNA pool were subject to test-PCR reactions monitored in real-time. Based on the obtained amplification curves, we determined the minimum number of cycles needed for subsequent PCR amplification of the pool to prevent the formation of by-products and artefacts (see Supplementary Figure S1 and “Methods” for details).

As outlined in Table 1, our selection strategy involved gradually increasing the selection pressure from round 2 (R02) onward by decreasing the concentration of input DNA (from 1.8 to 0.01 nmol), increasing the number of wash cycles (from one to five) and adding competitors in excess (BSA and salmon sperm DNA) to the binding reaction, while the number of target cells was kept constant (10^7^ cells). To eradicate nonspecific binders from the pool that bind to common and highly conserved cell-surface molecules, we introduced subtractive counter-selections early in SELEX round R03 to R06 against mixtures of ten *Enterococcus* spp. (*E. faecium, E. durans, E. hirae, E. gallinarum, E. casseliflavus, E. sulfureus, E. cecorum, E. avium, E. asini* and *E. mundtii*) and later in SELEX R09 against a mixture of seven non-*Enterococcus* spp. (*Streptococcus bovis, Streptococcus salivarius*, *Staphylococcus aureus*, *Escherichia coli*, *Citrobacter freundii*, *Klebsiella aerogenes* and *Pseudomonas aeruginosa*). Sequences that bound to these counter-target cells were removed before the incubation with *E. faecalis* cells (no amplification step in-between). We refrained from using heat-elution and/or purification steps to detach the bound ssDNA from *E. faecalis* target cells prior to amplification in order to minimize the loss of potential aptamers associated with such additional steps^35^. We did not observe detrimental effects of cells or cell components on the efficiency of the employed PCR assays.

**Table 1:** Selection conditions used for SELEX against *E. faecalis.*

SELEX round	ssDNA		Bacteria		Incubation procedure			Washing
	Input (nmol)	Conc. (nM)	Target	Total number of cells used	Temperature and time	Reaction volume (µL)	Competitors	Washes (mL)
R01	1.800	7200	*E. faecalis*	10^7^	21 °C, 30 min	250	–	1 × 1
R02	0.025	125	*E. faecalis*	10^7^	21 °C, 30 min	250	–	3 × 1
R03^**a**^	0.025	125	*E. faecalis*	10^7^	21 °C, 30 min	250	–	3 × 1
R04^**a**^	0.025	125	*E. faecalis*	10^7^	21 °C, 30 min	250	BSA, salmon sperm DNA	3 × 1
R05^**a**^	0.010	100	*E. faecalis*	10^7^	21 °C, 30 min	100	BSA, salmon sperm DNA	3 × 1
R06^**a**^	0.010	100	*E. faecalis*	10^7^	21 °C, 30 min	100	BSA, salmon sperm DNA	5 × 1
R07	0.010	100	*E. faecalis*	10^7^	21 °C, 30 min	100	BSA, salmon sperm DNA	5 × 1
R08	0.010	100	*E. faecalis*	10^7^	21 °C, 30 min	100	BSA, salmon sperm DNA	5 × 1
R09^b^	0.010	100	*E. faecalis*	10^7^	21 °C, 30 min	100	BSA, salmon sperm DNA	5 × 1
R10	0.010	100	*E. faecalis*	10^7^	21 °C, 30 min	100	BSA, salmon sperm DNA	5 × 1
R11	0.010	100	*E. faecalis*	10^7^	21 °C, 30 min	100	BSA, salmon sperm DNA	5 × 1

^a^Counter-selection performed before incubation with *E. faecalis* cells with a mixture of *E. faecium, E. durans, E. hirae, E. gallinarum, E. casseliflavus, E. sulfureus, E. cecorum, E. avium, E. asini* and *E. mundtii.*

^b^Counter-selection performed before incubation with *E. faecalis* cells with a mixture of *S. bovis, S. salivarius*, *S. aureus*, *E. coli* (NCTC 9001), *C. freundii*, *K. aerogenes* and *P. aeruginosa.*

### Monitoring the SELEX process by qPCR analyses

Most previous studies describing the selection of aptamers against bacterial cells applied spectrophotometric measurements (e.g., Nanodrop) to determine and monitor the proportion of ssDNA bound to the target cells and terminated the selection once a plateau was reached^17,36,37^. To monitor and assess the selection process in more detail, we used qPCR to quantify the cell-bound ssDNA pools from individual SELEX rounds and to determine the total amount of recovered ssDNA after incubation and washing. Cell-bound aptamer pools were then diluted and normalized (10^5^ ssDNA molecules per reaction) for remelting curve analysis to estimate changes in pool diversity based on double-stranded qPCR products. In parallel, dilutions of the original random unselected ssDNA library (100% diversity at different ssDNA concentrations) were analysed to determine the average melting temperature and to compare the melting profiles. All SELEX rounds were subjected to these qPCR-based analyses, except for R01. R01 was excluded as the entire volume of cell-bound ssDNA pool was PCR amplified for the next SELEX round to recover all sequences, which are all unique at this point of the selection^12,35^.

**Figure 2:**
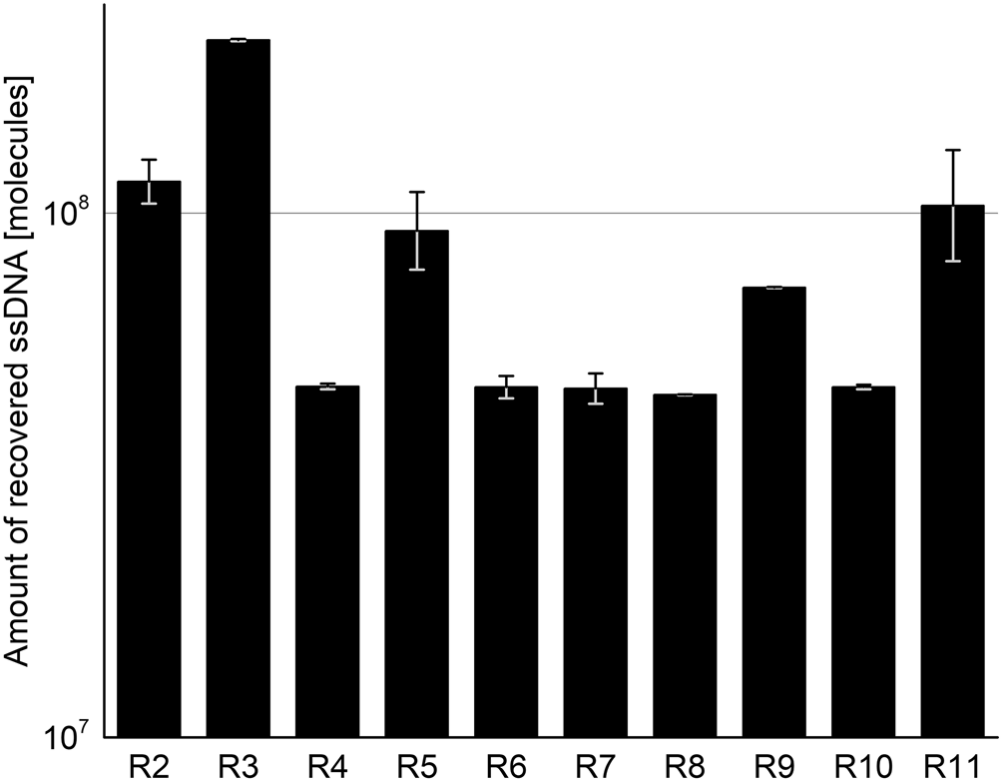
Total amounts of recovered ssDNA molecules from consecutive rounds of SELEX (R02–R11) measured by qPCR. To test for inhibitory effects, each sample was analysed undiluted and diluted 1:10. As no inhibitory effect was observed, the mean ssDNA quantity was calculated from both undiluted and diluted measurements. Error bars represent min. and max.

Based on the qPCR results (Fig. 2), we found that the mean total amount of ssDNA recovered from R02 was 1.1 × 10^8^ ssDNA molecules, which equals approximately 11 ssDNA molecules bound on average per *E. faecalis* cell. After eleven rounds of SELEX and with increased selection pressure and several counter-selections, the amount of cell-bound ssDNA and thus the number of ssDNA molecules bound to *E. faecalis* cells remained constant.

**Figure 3:**
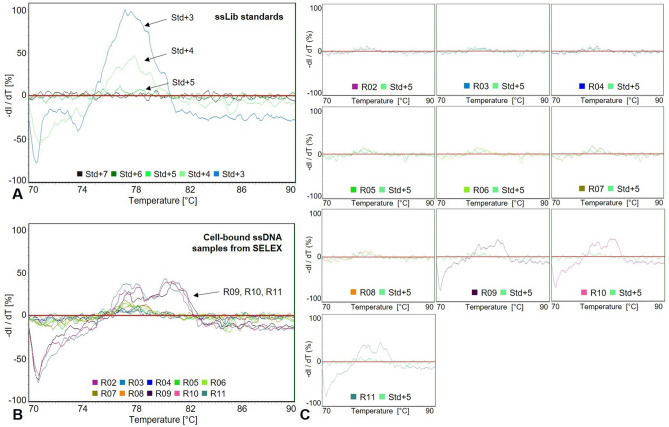
Monitoring the progression of the whole cell-SELEX process by qPCR-based remelting curve analysis. (**A**) Remelting curves from dilutions of the random ssDNA library ranging from 10^7^ (Std + 7) to 10^3^ (Std + 3) different molecules per reaction. (**B**) Remelting curves of cell-bound ssDNA pools from consecutive SELEX rounds (R02–R11) normalized to 10^5^ molecules per reaction. (**C**) Individual remelting curves of the cell-bound ssDNA pools compared to random ssDNA library dilution 10^5^ (Std + 5). Ascending melting peaks indicated the accumulation of sequences in the cell-bound ssDNA pools.

However, remelting curve analysis indicated that the diversity of these cell-bound sequences changed (Fig. 3). Based on the dilutions of the random ssDNA library we simulated enrichment to qualitatively assess the diversity of the ssDNA pools. As evident from Fig. 3A, highly diverse ssDNA pools with ≥ 10^5^ different sequences showed continuous melting behaviour without distinct melting temperatures (ssLib dilutions + 7, + 6 and + 5). On the other hand, less diverse pools with < 10^5^ different sequences (ssLib dilutions + 4, + 3) showed more discrete melting temperature ranges. This stems from a larger proportion of complementary sequences that form stable homo-duplexes after total denaturation and re-annealing at high temperatures (70 °C) when sequence diversity is low^31^. By comparing the remelting data of cell-bound ssDNA pools and the random (unselected) ssDNA library, we observed that distinct melting peaks appeared in SELEX R09 suggesting a drop in pool diversity and the emergence of potentially enriched sequences (Fig. 3B,C). From SELEX R09 to R11, melting peaks did not increase further, but changed their remelting shape, suggesting changes in sequence composition. In addition to melting peak height and shape, we also observed a shift towards higher melting temperatures. The average melting temperature of 77.6 °C (random ssDNA library, Fig. 3A) increased to 79.3 °C (R11, Fig. 3B). All these effects can be attributed to a change in pool composition and the accumulation of sequences^31^. Additional amplification and standard melting curves performed prior to remelting curve analysis (see “Methods” section for details) are given in Supplementary Figure S2. We ended the selection process after eleven rounds as further selection rounds are not only costly and time-consuming but can also make the selection prone to amplification errors or even the loss of aptamers^38^. To confirm the observation of sequence enrichment and to identify the sequences that bound to *E. faecalis* cells, we performed next-generation sequencing (NGS).

### NGS data analysis and assessment of the SELEX process

We performed NGS sequencing on an Illumina MiSeq sequencer to analyse the ssDNA pools from SELEX round R02–R11 and to identify their sequence composition. A total of 2,948,294 reads were successfully demultiplexed by the MiSeq and used as input for our analysis workflow. Adding sequencing data of the starting ssDNA library (R00) from another run, our data analysis pipeline started with 3,055,932 reads. Filtering for intact 5′ and 3′ constant regions and correct insert length (i.e., removing too short and too long reads) left us with 2,686,568 reads (87%). Discarding low quality reads (Phred Q-score ≤ 30) reduced this number to 2,094,663 reads (69%). Paired-end reads were merged as an additional quality control but had only minor impact on the number of individual reads, resulting in a total of 2,089,419 reads (68%), which were used for further analysis. After data pre-processing, sequences were tracked over successive SELEX rounds to assess the progression of the SELEX process and to verify the sequence enrichment indicated by qPCR-based remelting curve analysis. To this end, sequences were grouped into different categories according to their read numbers (i.e. frequency: 1–9, 10–99, 100–999, 1,000–9,999) and normalized to the total number of reads in each SELEX round (% of total reads). Moreover, we performed nucleotide composition analysis of the 40-nt random region to check the quality of starting ssDNA library (equal distribution of 25% A, T, C, G) and to investigate if the composition changed over the selection process.

**Figure 4:**
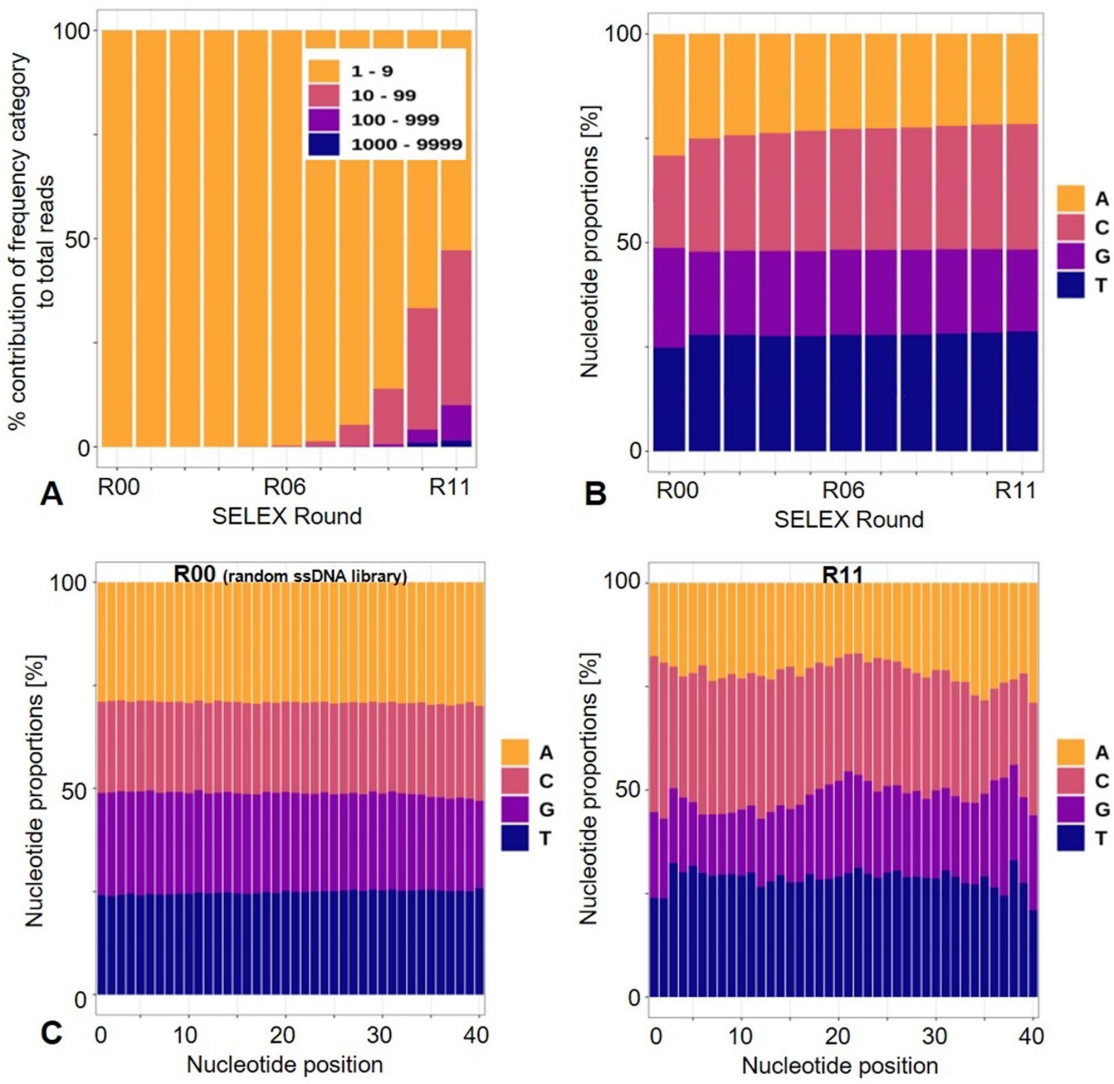
NGS analysis of the ssDNA population. (**A**) Proportion of reads (%) in the ssDNA pools from SELEX R02–R11 and the random ssDNA library (R00) that were counted 1–9, 10–99, 100–999, 1000–9999 times. (**B**) Changes in the nucleotide composition in the random regions over the selection rounds. (**C**) Distribution of nucleotides at each position of the 40-nt long random region of the random ssDNA library (R00) and the ssDNA pool of SELEX R11.

NGS analysis revealed that sequence enrichment was first observable from SELEX round R06 to R07 when sequences with > 10 reads appeared (Fig. 4A). From SELEX R07 onward, the proportion of unique sequences and sequences with < 10 reads, respectively, steadily decreased while sequences with 10–99 and 100–999 reads increased. In ssDNA pools of SELEX R10 and R11, we also found sequences with > 1000 reads (Fig. 4A). Moreover, we determined that nucleotide composition changed over the course of the selection. As shown in Fig. 4B, the average proportion of nucleotide bases in the random region of the starting random ssDNA library (R00) was biased towards adenine-rich sequences, while cytosine and guanine were slightly underrepresented (A: 29.1%, C: 22.1%, G: 23.9%, T: 24.8%). After eleven rounds of SELEX, however, cytosine- and thymine-rich sequences had increased by 8.0% and 3.8%, respectively (R11; A: 21.6%, C: 30.1%, G: 19.7%, T: 28.6%). Likewise, the nucleotide distribution at each position of the 40-nt randomized regions was altered in the ssDNA population from SELEX R11 (Fig. 4C).

Candidate sequences for further testing were chosen from a compiled sequence table in which all sequences were ranked based on their frequency in R11 and tracked over the preceding rounds of SELEX. In addition, lists of the top 1000 most frequent sequences of each SELEX round were created (Supplementary Material S1). The following criteria were used for selecting sequences: sequence frequency, appearance in early rounds, sequence enrichment in rounds with counter-selection and increased selection pressure, as well as on predicted secondary structure and minimal free energy. Based on these criteria, we selected and chemically synthesized 16 aptamer candidate sequences for testing (Table 2). Their predicted secondary structures and minimal free energies are given in Supplementary Figure S3.

**Table 2:** Full-length sequences of aptamer candidates from NGS analysis including their rank number in the top 1000 reads of the final SELEX round (R11).

Name	Sequence (5′–3′)	Rank
EF501	*TAGGGAAGAGAAGGACATATGAT*TTTCTCAACGGGACCATCACTTACCTCAAGTACTTGGACG*TTGACTAGTACATGACCACTTGA*	#1
EF502	*TAGGGAAGAGAAGGACATATGAT*CCGGCTATCTCCCTACCGTGGCCGAGTACCTCAAACGTTT*TTGACTAGTACATGACCACTTGA*	#3
EF503	*TAGGGAAGAGAAGGACATATGAT*GTCAACTCATTTATGGTGCTCCTCGTACCTCAGGTGGTTA*TTGACTAGTACATGACCACTTGA*	#2
EF504	*TAGGGAAGAGAAGGACATATGAT*GGCCATACCTCGTGCCTTCTGTGATCATCTCTATCAATTG*TTGACTAGTACATGACCACTTGA*	#8
EF505	*TAGGGAAGAGAAGGACATATGAT*CCTCTCTCTTACTGCTACTGGGCAGGGTACTCAATTACGT*TTGACTAGTACATGACCACTTGA*	#10
EF506	*TAGGGAAGAGAAGGACATATGAT*CGGTCCCGACTCAATATTGTTCCCTCCCCTTATCAGGCGG*TTGACTAGTACATGACCACTTGA*	#11
EF507	*TAGGGAAGAGAAGGACATATGAT*TCCTCTAATCAACTCTATGCCTTATCCCCTTGGTCAGGAC*TTGACTAGTACATGACCACTTGA*	#12
EF508	*TAGGGAAGAGAAGGACATATGAT*ACTGGCCTTGACACCCTGTTGTGGCTTGATGACAATAACA*TTGACTAGTACATGACCACTTGA*	#21
EF509	*TAGGGAAGAGAAGGACATATGAT*CCTCACTCTTGACCCAAAGTGCATGCTCTATTCATTCGGA*TTGACTAGTACATGACCACTTGA*	#58
EF510	*TAGGGAAGAGAAGGACATATGAT*GCTTCTGTGCACATTAAGGCACTCGTCTTCACTGTGGTTC*TTGACTAGTACATGACCACTTGA*	#86
EF511	*TAGGGAAGAGAAGGACATATGAT*CCTAACTCACTTACCAGCACGAGGTGCCTGTACCATCAAT*TTGACTAGTACATGACCACTTGA*	#78
EF512	*TAGGGAAGAGAAGGACATATGAT*CTCTCATCACAGGAATTTGAATTTCCCTTGTGGACAGTAA*TTGACTAGTACATGACCACTTGA*	#74
EF513	*TAGGGAAGAGAAGGACATATGAT*GATGTGAATTCCGTCCCTTGGTCAGACACTTCAACACCGG*TTGACTAGTACATGACCACTTGA*	#216
EF514	*TAGGGAAGAGAAGGACATATGAT*TCTCGACGCTATGATCAAGACGCAGTATGATGGCACATCA*TTGACTAGTACATGACCACTTGA*	#235
EF515	*TAGGGAAGAGAAGGACATATGAT*TTAACCCTCATTTAATGGCCGCGTCAATCCGCAAAGGGTC*TTGACTAGTACATGACCACTTGA*	> #1000
EF516	*TAGGGAAGAGAAGGACATATGAT*TTCCTTCGCAGGACACCGATGGCCAGGCGCGAGTCAATAT*TTGACTAGTACATGACCACTTGA*	> #1000

Constant primer regions are written italic.

### Aptamer identification

**Figure 5:**
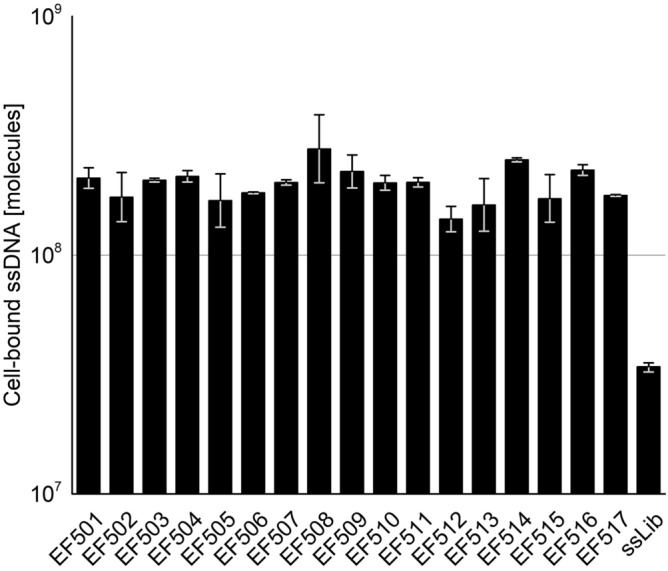
Results from initial screening of aptamer candidate sequences EF501 to EF516 based on qPCR analysis. Each aptamer was incubated with 10^7^ *E. faecalis* cells in 100 µL binding buffer at a final aptamer concentration of 100 nM for 30 min at 21 °C and mild shaking. After washing three times by centrifugation, the amount of cell-bound aptamer was quantified via qPCR. Reactions with the random library (ssLib), without cells (blank samples) and without ssDNA (cells only, negative control) were run in parallel. Two biological replicates were performed for each sample. Reactions were judged free of contamination based on negative controls. Data are represented as the geometric mean of two biological replicates, after subtraction of blank samples. Error bars represent min. and max.

A series of binding assays based on qPCR analysis was performed (1) to screen the putative full-length candidate sequences in terms of their binding properties and (2) to select the most promising ones for further analysis. To this end, each candidate was incubated at a final concentration of 100 nM with 10^7^ *E. faecalis* target cells in binding buffer. Several controls were carried out in parallel. First, blank reactions were performed without bacterial cells to determine the amount of non-specific background binding and to exclude differences in the observed binding that is possibly based on the sequence’s tendency to adhere to plastic tube surfaces. Measured aptamer quantities from these control samples were subtracted from positive samples (i.e., *E. faecalis* cells + ssDNA). Second, the random ssDNA library was assessed in parallel to compare the interaction behaviour. Furthermore, reactions with *E. faecalis* cells only (no ssDNA added) were performed to check for contamination. Two biological replicates were performed for each candidate tested. Results indicated that all tested candidates exhibited similar levels of binding to *E. faecalis* cells, which were on average 7-times higher than that of the random ssDNA library (Fig. 5). Among all 16 tested sequences, aptamer candidate EF508 displayed the highest mean cell-bound ssDNA quantity.

**Figure 6:**
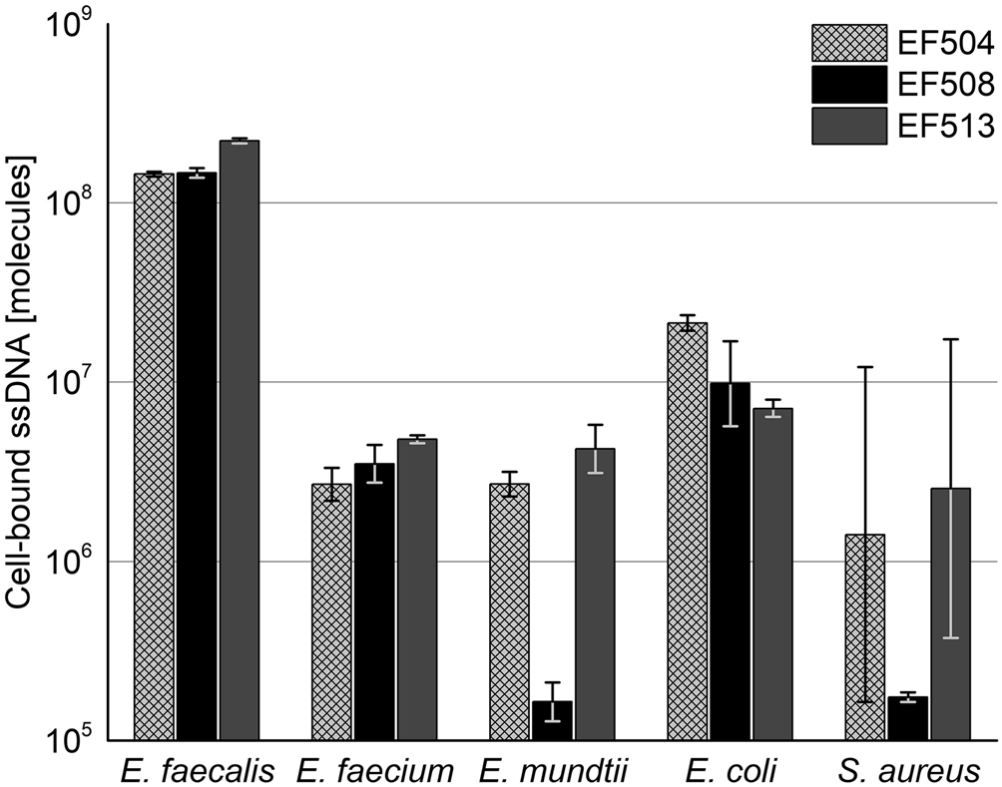
Binding assay results from aptamer candidates EF504, EF508 and EF513 evaluating the specificity of the sequences towards non-target species *Enterococcus faecium, Enterococcus mundtii, Escherichia coli* NCTC 9001 and *Staphylococcus aureus.* 10^7^ cells were used per reaction. All aptamers were tested in a final concentration of 100 nM. Blank reactions (without cells) and negative controls (without ssDNA) were run in parallel as a control. Two biological replicates were performed for each sample and the amount of cell-bound aptamer determined via qPCR. Based on negative controls, reactions were judged free of contamination. Data are represented as the geometric mean of two biological replicates, after subtraction of blank samples (reactions without cells). Error bars represent min. and max.

Based on the enrichment trajectories of the candidates, we tested the specificity of aptamer candidate EF508 and two other candidates EF504 and EF513 with higher and lower sequencing reads (Supplementary Table S1), respectively, on a small subset of non-target cells, comprising two *Enterococcus* spp. (*E. faecium, E. mundtii)* and two non-*Enterococcus* spp. (Gram-negative *Escherichia coli* NCTC9001 and Gram-positive *Staphylococcus aureus*). All four species were used for counter-selection in SELEX. The binding experiment was performed as described before using 100 nM aptamer and 10^7^ bacteria cells. We found that all three aptamer candidates displayed higher binding to *E. faecalis* cells than to any of the tested non-target species (Fig. 6). However, we observed distinct differences in the level of specificity of the three aptamers. Based on these screening results and NGS data that showed that aptamer EF508 appeared early from R05 onwards (Supplementary Table S1), albeit at low read number, EF508 was further characterized in more detail.

### Aptamer binding affinity

**Figure 7:**
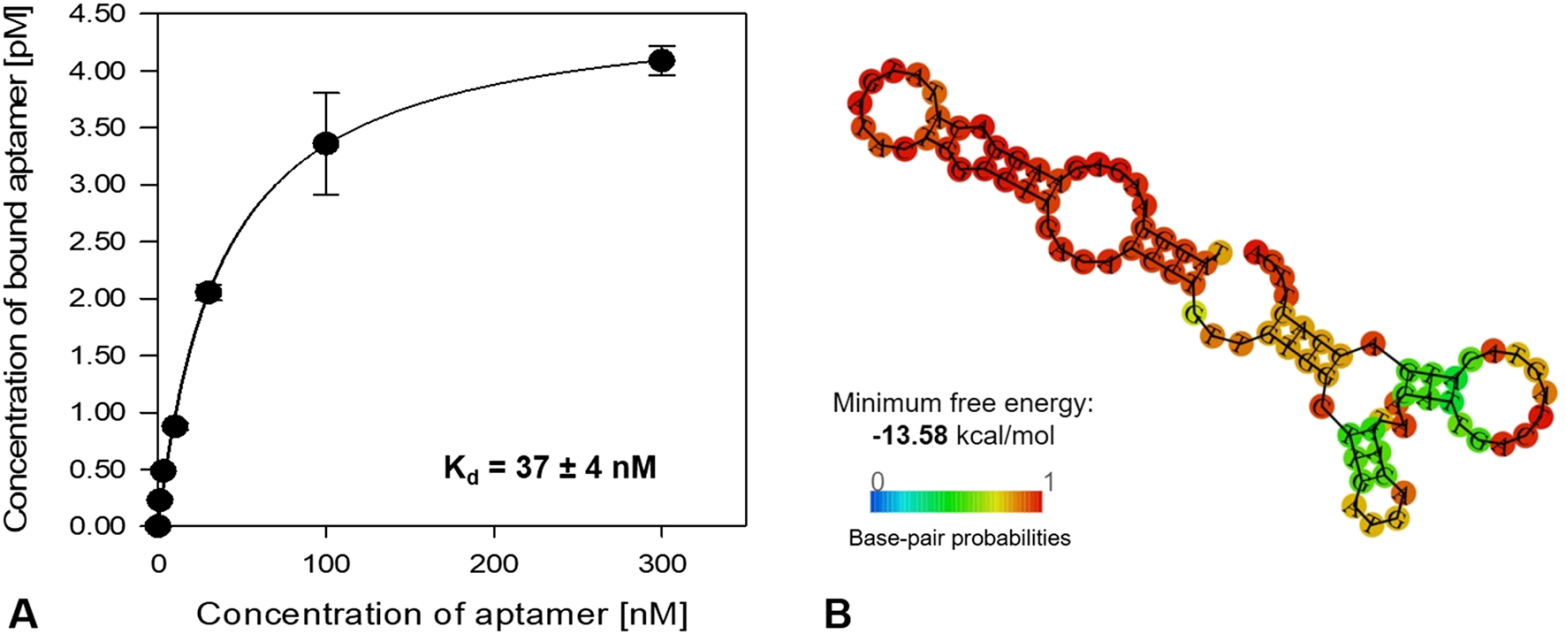
(**A**) Binding saturation curve of aptamer EF508 to *E. faecalis* cells. Cells were incubated with increasing concentrations of aptamer (0, 1, 3, 10, 30, 100 and 300 nM) and measured by qPCR. Data points represent the mean of three biological replicates. A non-linear regression curve was fitted to the data using SigmaPlot version 14.0. The dissociation constant (K_D_) is shown as the mean ± SD. (**B**) Predicted secondary structure and minimal free energy of aptamer EF508 using RNAfold software.

The binding affinity of aptamer EF508 was assessed by incubating 10^7^ *E. faecalis* cells with different aptamer concentrations ranging from 0 to 300 nM. Data obtained from qPCR analysis showed a typical saturation curve, which were fitted by non-linear regression analysis (Fig. 7A). This indicates that the aptamer interacts with *E. faecalis* cells in a concentration-dependent manner. The determined dissociation constant (K_D_) was 37 ± 4 nM, which is comparable to previously reported K_D_ values of aptamers against bacteria cells that typically range between 10 and 200 nM^36,37,39^. The predicted most probable secondary structure of aptamer EF508 by RNAfold, with stable and defined stem-loop structures, is shown in Fig. 7B.

### Aptamer specificity and cross-reactivity towards bacteria species

In order to more broadly assess the specificity of the aptamer EF508, a total of 20 bacterial species were tested. The sample set consisted of ten *Enterococcus* spp., including *E. faecium, E. durans, E. hirae, E. gallinarum, E. casseliflavus, E. sulfureus, E. cecorum, E. avium, E. asini* and *E. mundtii*. Likewise, we tested five Gram-positive species, which are phylogenetically closely related to enterococci, namely *Streptococcus bovis, Streptococcus salivarius*, *Lactococcus lactis* and *Staphylococcus aureus*, as well as five Gram-negative species which are often found in fecally polluted waters, including three *Escherichia coli* strains (NCTC 9001, ATCC 25922, ATCC 8739), *Citrobacter freundii*, *Klebsiella aerogenes* and *Pseudomonas aeruginosa.* All these species had been used for counter-selection in SELEX, except for *E. coli* strains ATCC 25922 and ATCC 8739 and *L. lactis*. Each species was tested individually using 10^7^ cells per reaction and 100 nM aptamer. Three biological replicates were performed and analysed with qPCR.

**Figure 8:**
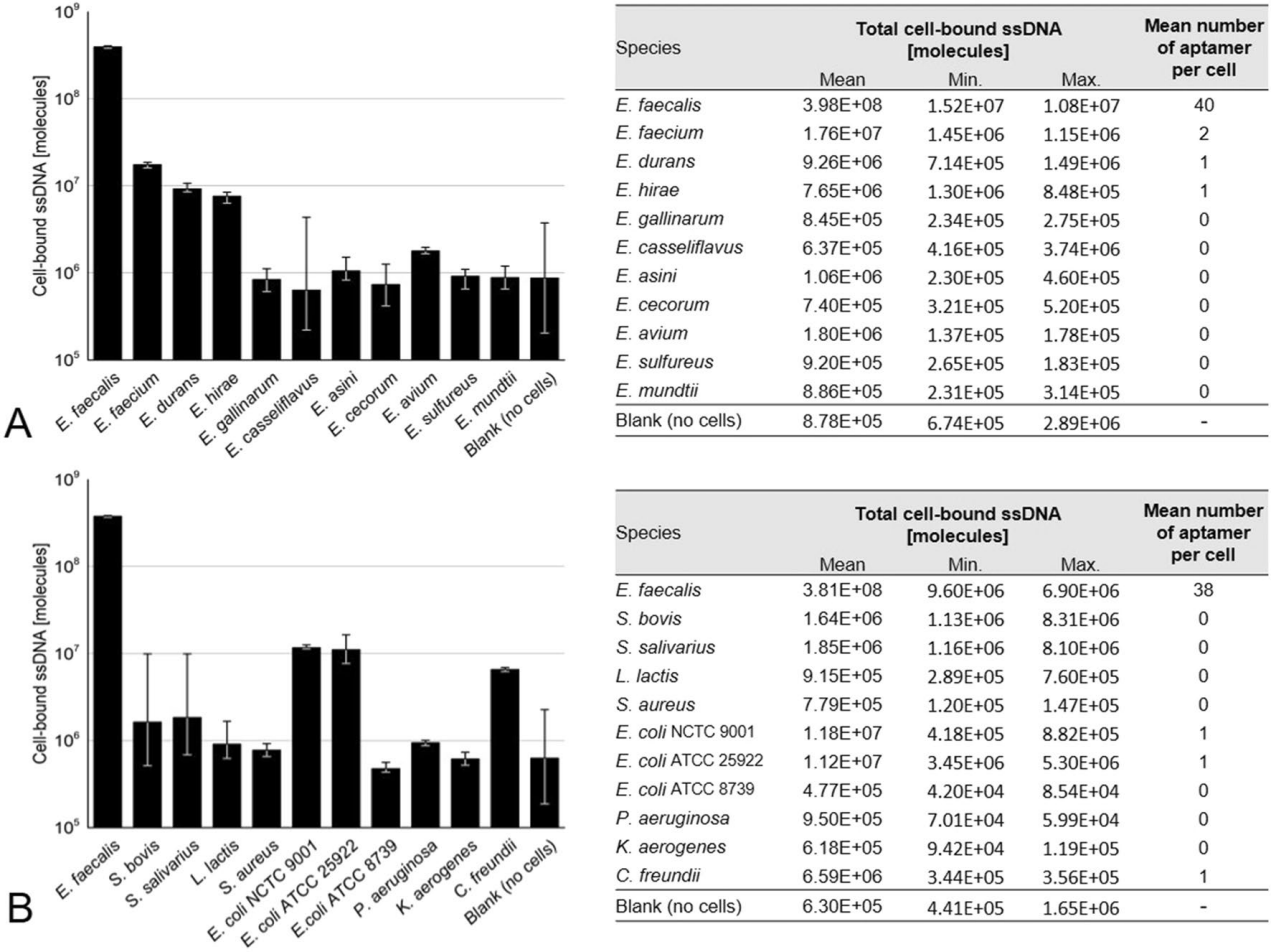
Specificity of aptamer EF508 evaluated with (**A**) ten *Enterococcus* spp. and (**B**) ten non-*Enterococcus* spp. *E. faecalis* target cells were tested in parallel. Binding assays were performed with 100 nM of aptamer and 10^7^ bacteria cells. Blank reactions (without cells, only aptamer) and negative controls (without ssDNA) were run in parallel as a control. Three biological replicates were performed for each sample and the amount of cell-bound aptamer determined via qPCR. Based on negative controls, reactions were judged free of contamination. Data are represented as the geometric mean of three biological replicates. Error bars represent min. and max. Based on the amount of recovered ssDNA, the average number of aptamers bound per cell was estimated.

Based on qPCR results, aptamer EF508 showed a high degree of specificity (Fig. 8). It can not only discriminate *E. faecalis* from other *Enterococcus* spp., but also from both Gram-positive and Gram-negative species. Based on the calculated number of bound aptamers per cell, less than one aptamer molecule on average was bound to non-target cells. Likewise, no apparent binding to *E. coli* strains ATCC 25922 and ATCC 8739 as well as *L. lactis* DSM 20481 was observed, even though these strains were not used as counter-targets in SELEX. Comparing the total measured ssDNA quantities, we observed a more than 20- to 800-fold higher binding to *E. faecalis* target cells than to non-target cells, which to our knowledge is higher than previously reported for DNA aptamers that were selected in bacterial cell SELEX experiments. Typically, five- to ten-fold differences were observed in specificity tests that comprised smaller sample sets (three to twelve tested species)^22, 36^. These differences could stem from the high sensitivity of qPCR-based measurements, compared to e.g. fluorescence spectroscopy- or flow cytometry-based analyses. However, Marton et al.^22^ conducted qPCR-based specificity tests and also reported only a ten-fold higher binding of their aptamer to *E. coli* target cells than to twelve other bacterial species tested^22^.

**Figure 9:**
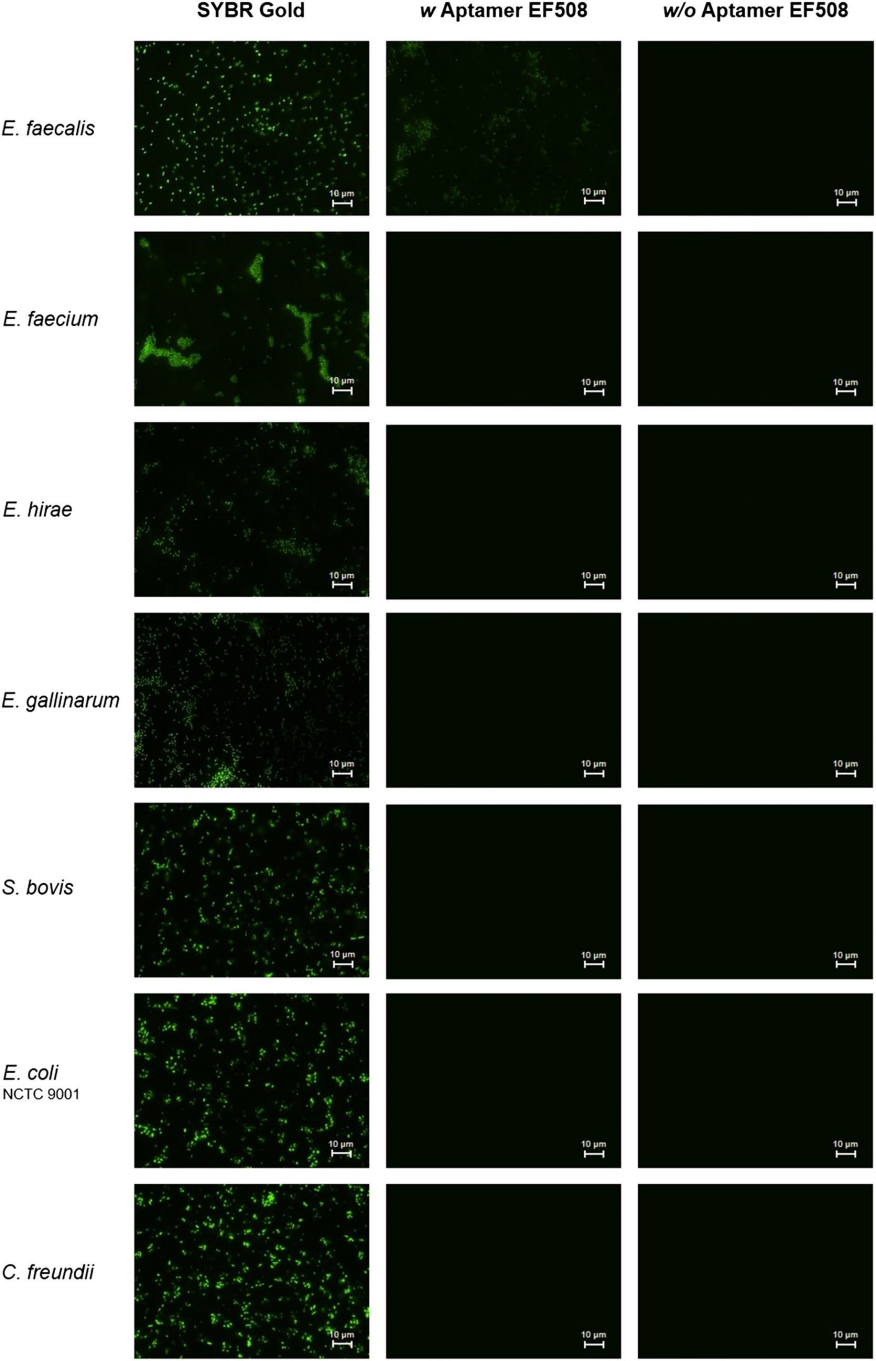
Epifluorescence microscopy image of *E. faecalis* target cells and six bacterial non-target species stained with SYBR Gold (left column), 100 nM of 5′-FAM labelled aptamer EF508 (middle column) and without any staining (right column). Experiments were carried out with 10^7^ cells collected on a 0.2 µm membrane filter for microscopic observation. Magnification: 1000 ×, Exposure time: 100 ms. White scale bars denote 10 µm.

Specific binding of the aptamer was also visually confirmed and qualitatively assessed by epifluorescence microscopy on a subset of bacterial stains, including *E. faecalis* target cells and six non-target cells (*E. faecium*, *E. hirae*, *E. gallinarum*, *S. bovis*, *E. coli* NCTC 9001 and *C. freundii*). Each species was tested individually using 10^7^ cells that were incubated with 100 nM of 5′-FAM labelled aptamer EF508. In parallel, experiments were performed without aptamer (binding buffer used instead of aptamer). These blank reactions were either subjected to SYBR Gold staining serving as control and reference for direct cell detection approaches or were left untreated to control for any fluorescence signals caused by autofluorescence of the bacterial cells. As shown in Fig. 9, *E. faecalis* target cells incubated with the aptamer induced fluorescence reflecting the morphology of the bacteria, albeit at lower intensity than the non-selective SYBR Gold fluorescence dye. In contrast, non-target cells incubated with 5′-FAM-labelled aptamer showed no fluorescence (Fig. 9).

Overall, the binding preference of aptamer EF508 suggests that the selected candidate binds to a molecular target that is predominantly expressed on the cell surface of type strain *E. faecalis*. However, further studies would be needed to identify this unknown cell-surface epitope, e.g. using pull-down experiments from the cell followed by LC–MS/MS analysis^40^. Likewise, comprehensive studies including environmental isolates, establishing limits of detection and testing different sample matrices would be necessary to ensure the functionality and robustness of the aptamer in future downstream diagnostic applications. Depending on the field of application (e.g. water quality testing), signal enhancement strategies might be needed for establishing future aptamer-based epifluorescence microscopy methods (e.g. using quantum dot labelling^41^). Based on our results, however, we anticipate that aptamer EF508 is a promising candidate for any such endeavours.

## Conclusion

In this study, we used a customized aptamer discovery approach to select and identify DNA aptamers for *E. faecalis* cells. We applied state-of-the-art applications of qPCR as tools for optimizing the PCR amplification step of the SELEX procedure and for assessing the overall selection process in-line while it is progressing. We also showed that measuring the ssDNA pools size during bacterial cell-SELEX may not always be informative. Solely based on amounts of cell-bound ssDNA measured by qPCR, we were unable to observe a selection progress. Yet, sequence diversity changed as indicated by remelting curve analysis of qPCR products and confirmed by NGS analysis. Thus, we conclude that remelting curve analysis is a highly useful complementary tool to gain information on sequence enrichment and to rationally decide whether a bacterial cell-SELEX experiment should be continued, sequenced or rather be aborted. Due to next-generation sequencing and data analysis, we were able to trace the population dynamics over the selection procedure based on ssDNA pools from several rounds that were barcoded and sequenced in parallel. The obtained data illustrated that NGS data are vital in identifying bacterial cell-aptamer candidates. Based on this new and combined approach, we identified aptamer EF508, which made up 0.394% of the final pool population and which displayed high binding affinity in the nanomolar range and high specificity to *E. faecalis* target cells. Even though future work will be required to fully characterize and test this novel binding ligand, we conclude that the aptamer has significant potential to be used for diagnostic applications.

## Methods

### Bacterial strains

Type strain *Enterococcus faecalis* (*E. faecalis*) DSM-20478 was used as target for the in vitro aptamer selection process. For counter-selection and characterization studies the following organisms were used: *E. faecium* (DSM-20477), *E. durans* (DSM-20633), *E. hirae* (DSM-20160), *E. gallinarum* (DSM-20628), *E. casseliflavus* (DSM-20680), *E. mundtii* (DSM-4838), *E. sulfureus* (DSM-6905), *E. cecorum* (DSM-20682), *E. avium* (DSM-20679), *E. asini* (DSM-11492), *Streptococcus bovis* (DSM-20480), *Streptococcus salivarius* (DSM-20560), *Lactococcus lactis* (DSM-20481), *Staphylococcus aureus* (DSM-20232), three strains of *Escherichia coli* (NCTC 9001, ATCC 25922, ATCC 6633), *Citrobacter freundii* (DSM-30039), *Klebsiella aerogenes* (NCTC 9528) and *Pseudomonas aeruginosa* (NCTC 10662). The bacterial strains were purchased from the German Collection of Microorganisms and Cell Cultures (DSMZ), Braunschweig, Germany and were cultivated under aerobic conditions until exponential growth phase. *Enterococcus* spp. and *Streptococcus* spp. were grown in tryptic soy broth, whereas all other species were cultivated in Brain Heart Infusion. Detailed information on the preparation of stock solutions and the cultivation conditions are given in the Supplementary Information S1.

Unless otherwise noted, aliquots of the prepared stock solutions were used for SELEX and binding assay experiments. Before use, the cells were washed two times with 1 mL of 0.2 µm-sterile filtered 1 × PBS, pH 7.4 (137 mM NaCl, 2.7 mM KCl, 8 mM Na_2_HPO_4_, and 2 mM KH_2_PO_4_; Thermo Fisher Scientific, AM9625) by centrifugation at 13,000×*g* for 10 min. The cell pellet was finally resuspended in aptamer binding buffer (1 × PBS, pH 7.4 with 0.05% [v/v] Tween20; 0.2 µm-sterile filtered).

### ssDNA library and primers

The synthetic ssDNA library consisted of a central 40-nt randomized region flanked on either side with 23-nt constant primer binding regions for PCR (5′-TAG GGA AGA GAA GGA CAT ATG AT—N_40_—TTG ACT AGT ACA TGA CCA CTT GA-3′). It was chemically synthesized and HPLC-purified by Integrated DNA Technologies (Coralville, USA). Unmodified forward primer (5′-TAG GGA AGA GAA GGA CAT ATG AT), unmodified reverse primer (5′-TCA AGT GGT CAT GTA CTA GTC AA-3′) and 5′-phosphorylated reverse primer (5′-Phos-TCA AGT GGT CAT GTA CTA GTC AA-3′) were purchased from Eurofins MWG Operon (Ebersberg, Germany). The synthetic ssDNA library and the primers were dissolved in nuclease-free water (Roth, Germany) to a final concentration of 100 µM.

### Bacterial whole cell-SELEX process

In total, eleven SELEX rounds were performed (Table 1). For the first round of SELEX, 1.8 nmol of the ssDNA library (~ 10^15^ molecules) were prepared in 50 µL binding buffer, heated at 95 °C for 5 min and kept at 21 °C for 30 min to allow the formation of folded DNA structures. The folded ssDNA library was mixed and incubated with 10^7^ washed *E. faecalis* cells in a final reaction volume of 250 µL binding buffer at 21 °C for 30 min with moderate shaking at 550 rpm on an Eppendorf thermomixer. After the binding reaction, the cells were collected by centrifugation for 10 min at 13,000×*g*. The supernatant containing unbound sequences was discarded and the cell pellet washed once by resuspending the cells in 1 mL binding buffer. After centrifugation for 10 min at 13,000×*g*, the bacteria-aptamer complexes were recovered by discarding the binding buffer and resuspending the cell pellet in 50 µL nuclease-free water. This fraction is referred to as “cell-bound ssDNA pool”. To eliminate sequences that bind to the tube walls, the mixture was transferred into a fresh 1.5 mL Eppendorf DNA LoBind tube (VWR, Germany). Heat-elution and/or purification steps of cell-bound ssDNA were omitted to minimize the loss of potential aptamers. Negative controls consisting of cells without added DNA were run in parallel throughout the entire procedure of incubation, washing and resuspension to check for cross-contamination.

In the first SELEX round, the entire cell-bound ssDNA pool (50 µL) was subject to preparative PCR, whereas in subsequent rounds approximately 15 µL of the cell-bound ssDNA pool was amplified by preparative PCR. PCR amplification of the cell-bound ssDNA pool was performed in a reaction volume of 25 µL containing 1 × Q5 High-Fidelity Master Mix (New England Biolabs, Germany), 1 µM forward primer, 1 µM phosphorylated reverse primer, and 1.0 µL of DNA template (cell-bound ssDNA; in the first round 5.0 µL DNA template were used for each reaction). All PCR reactions were run on a T100 Thermal Cycler (BioRad, Germany) according to the following thermal cycling conditions: an initial step of 3 min at 95 °C, followed by 15 s at 95 °C, 15 s at 55 °C, 15 s at 72 °C and a final extension step at 2 min at 72 °C. To avoid by-product formation, the number of PCR cycles was optimized for every round of SELEX as follows: prior to preparative PCR, a real-time monitored test-PCR reaction including 1 × EvaGreen fluorescence dye (Biotium/VWR, Germany) was performed on a Mastercycler ep realplex Real-time PCR system (Eppendorf, Hamburg, Germany). The cycle number before the amplification curve reached the fluorescence maximum was then used for subsequent preparative PCR amplification of cell-bound ssDNA fractions (typically between 20 and 25 cycles, Supplementary Figure S1).

To recover the ssDNA pool for subsequent selection rounds, PCR products were rendered single-stranded by Lambda-Exonuclease digestion^42^. To that end, all preparative amplification reactions were pooled, and PCR products were separated from primers and other reaction components using the Monarch PCR and DNA Cleanup Kit (New England Biolabs, Ipswich, USA). Two µg of purified dsDNA were then incubated with 5 U of Lambda Exonuclease (New England Biolabs, Ipswich, USA) in a total reaction volume of 50 µL at 37 °C for 60 min (four reactions with 2 µg of purified dsDNA each). The reactions were terminated by heat inactivation of the enzyme at 80 °C for 10 min. After ssDNA clean-up and concentration with the Monarch PCR and DNA Cleanup Kit, the recovered ssDNA was quantified via the NanoDrop One (Thermo Fisher Scientific, Germany). Aliquots of the preparative PCR product, the final enzyme digestion mixture and the purified ssDNA were analysed on a 4% agarose gel stained with SYBR Gold to check the size of the dsDNA and ssDNA products of each round, respectively.

Subsequent selection rounds were performed with decreasing amounts of input ssDNA (from 1.8 to 0.01 nmol), increasing number of washing cycles (from one to five washing cycles) and the addition of competitors, such as 0.5 µg µL^−1^ BSA (bovine serum albumin) and 0.25 µg µL^−1^ salmon sperm DNA in the binding buffer during incubation. To eliminate non-specific binding sequences from the ssDNA pool, counter-selections with mixtures of non-target bacteria were carried out before incubation with *E. faecalis* target cells as follows: ssDNA pools were first incubated with counter-target cells in binding buffer for 30 min at 21 °C and then the unbound ssDNA in the supernatant was withdrawn by centrifugation and incubated with 10^7^ *E. faecalis* cells. In rounds R03 to R06, ssDNA pools were counter-selected with a defined mixture of ten enterococcal non-target cells, namely *E. faecium, E. durans, E. hirae, E. gallinarum, E. casseliflavus, E. sulfureus, E. cecorum, E. avium, E. asini* and *E. mundtii* (10^7^ cells each, total of ~ 10^8^ cells). In round 9, ssDNA pools were counter-selected with a defined mixture of seven non-enterococcal non-target cells, namely *S. bovis, S. salivarius, S. aureus, E. coli (NCTC 9001), C. freundii, K. aerogenes* and *P. aeruginosa* (10^7^ cells each, ~ 8*10^7^ cells). The SELEX conditions used to isolate aptamers binding to *E. faecalis* cells are summarized in Table 1.

### Quantitative PCR

The total ssDNA quantities during SELEX and characterization studies, respectively, were measured via quantitative PCR (qPCR). All qPCR reactions were performed in a total reaction volume of 15 µL containing 1 × Kapa SYBR Fast (PeqLab, Germany), 500 nM unmodified primers, and 1.0 µL of cell-bound ssDNA pool. The amplification reactions were run on a Mastercycler ep realplex Real-time PCR system (Eppendorf, Germany) according to the following thermal cycling conditions: an initial step of 3 min at 95 °C, followed by 35 cycles of 15 s at 95 °C and 20 s at 62 °C. All amplification reactions were performed in duplicates, including no-template controls (NTC) in each run to check for contamination. Quantification was based on dilutions of the random synthetic ssDNA library. Standard dilutions were prepared in an unspecific background of 500 µg L^−1^ poly(dI-dC) (Roche Diagnostics, Germany) to avoid adsorption of ssDNA onto the reaction tubes at lower concentrations. A total of six tenfold serial dilutions of standard (10^3^–10^8^ ssDNA copies per reaction) were included in each qPCR run. Unless otherwise noted, samples were measured undiluted and diluted 1:10 in 10 mM of Tris–EDTA buffer (pH 8.5) and were judged free of inhibition if concentrations in the dilutions matched.

### Remelting curve analysis

To monitor the progress of the in vitro selection process, remelting curve analysis of PCR amplicons was performed as described earlier^31,32^ with several modifications. Reactions were performed in a total volume of 25 µL containing 1 × Q5 High-Fidelity Master Mix, 1 µM unmodified primers, 1 × EvaGreen dye and 1.0 µL of cell-bound ssDNA pool (normalized based on qPCR results to 10^5^ ssDNA molecules per reaction). Cycling conditions were 3 min at 95 °C, followed by 35 cycles at 15 s at 95 °C, 15 s at 55 °C and 15 s at 72 °C. After amplification, two melting curve analyses were performed (referred to as “standard melting curves” and “remelting curves”). The first standard melting curve protocol consisted of 15 s at 95 °C and reannealing for 15 s at 60 °C before heating to 95 °C in increments of 0.03 °C s^−1^. The second remelting curve protocol followed the same conditions, except reannealing was performed for 15 s at 70 °C before heating to 90 °C. Remelting curve analysis was performed with all cell-bound ssDNA pools from SELEX (R02-R11) and dilutions of the synthetic ssDNA library (10^7^–10^3^ ssDNA molecules per reaction). The ssDNA library dilution 10^5^ was used as a diversity standard representing maximum diversity to estimate sequence enrichment via remelting curves.

### Next-generation sequencing

The cell-bound ssDNA pools from SELEX R02 – R11 were prepared for next-generation sequencing (NGS) analysis following a modified 16S rRNA amplicon sequencing library preparation procedure^43^. In brief, a two-step PCR procedure was used to attach sequencing primer regions, barcodes and flow-cell adapters (Supplementary Figure S6). Primer sequences are given in Supplementary Table S3 and an overview of the resulting amplicons is shown in Supplementary Figure S7. The first NGS-PCR used primers (NGS_PCR#1_forward and NGS_PCR#1_reverse) that consisted of SELEX-specific sequences (complementary to the constant primer binding regions of the ssDNA pools) and overhang sequences that included Illumina’s sequencing primer sites. PCR reactions were carried out in a total volume of 25 µL containing 1 × Q5 High-Fidelity Master Mix, 0.5 µM of NGS_PCR#1 primers and equal amounts of ssDNA (10^6^ molecules per reaction). Cycling conditions were 3 min at 95 °C, followed by 22 cycles of 15 s at 95 °C, 15 s at 55 °C, 15 s at 72 °C and a final extension step for 2 min at 72 °C. The optimal number of cycles for the first NGS-PCR (22 cycles) was also determined by test-PCR reactions but using NGS_PCR#1 primers. After the first NGS-PCR, the PCR products were checked on a 3% agarose gel and were purified with the Agencourt AMPure XP Purification system (Beckman Coulter). The second NGS-PCR used primers (NGS_PCR#2_forward and NGS_PCR#2_reverse) that consisted of complementary overlapping sequences, barcodes (i7 and i5 indexes) and adapter sequences to attach the oligonucleotides onto the flow cell. PCR reactions were carried out again in a total volume of 25 µL containing 1 × Q5 High-Fidelity Master Mix, 1 µM of NGS_PCR#2 primers and 2.5 µL of the purified first NGS-PCR product. Cycling conditions were 30 s at 95 °C, followed by 6 cycles of 10 s at 95 °C, 30 s at 55 °C, 30 s at 72 °C and a final extension step for 2 min at 72 °C. The second NGS-PCR was performed with a very limited number of cycles. Six cycles were sufficient to attach barcodes and flow cell adapters and to visualize the products on an agarose gel. The PCR products were purified as described before with the Agencourt AMPure XP Purification system. The DNA concentration of the final products was then determined with the QuantiFluor dsDNA system (Promega, Germany) and the GloMax-Multi + fluorometer (Promega, Germany) according to the manufacturer’s instructions. All ten samples (R02–R11) were mixed in equimolar ratio to a final concentration of 4 nM. The final NGS library was clustered at 8 pM with 10% of 8 pM PhiX added to the run. Sequencing was done on the Illumina MiSeq platform using the MiSeq Reagent Micro Kit v2 (300 cycles) in paired-end mode. Sequencing data were demultiplexed using bcl-2fastq2 v2.20.

### Sequencing data analysis

Analysis of the raw NGS-data was accomplished with a workflow implemented in Nextflow 19.10.0^44^ (illustrated in Supplementary Figure S8). Data were pre-processed using cutadapt 2.4^45^ to identify and cut the constant 5′ and 3′ SELEX primer binding regions. Every sequence had to consist of the 5′ end constant region ‘TAGGGAAGAGAAGGACATATGAT’, a random region of 40 ± 3 nt length, and the 3′ end constant region ‘TTGACTAGTACATGACCACTTGA’. An error rate of 20% (≤ 4 nt mismatches) within the constant regions was allowed. Sequences not fulfilling these conditions were discarded. Next, fastp 0.20.0^46^ was used to filter sequences by quality score, discarding all sequences of an overall Phred quality score ≤ 30. Fastp was also used for merging forward and reverse NGS-reads using the default parameters (5 mismatches allowed, min. 30 nt overlap). Log files from cutadapt and fastp were graphically processed using MultiQC v1.9^47^.

The pre-processed sequences were dereplicated using a custom counting script SELEXderep. The output of SELEXderep was used to determine every sequence’s reads per million total sequences (RPM) per round and to determine the ratio of unique to duplicate sequences of every round. Aptamers for further analysis were chosen based on RPM counts, enichment and structural properties of the aptamers, which were determined using RNAfold webserver v2.4.13 (https://rna.tbi.univie.ac.at/cgi-bin/RNAWebSuite/RNAfold.cgi)^48^. The custom script SELEXntcomposition was programmed to calculate the nucleotide composition of every round and for every position. Analysis was done using R 3.6.2 and Python 3.7. The following R libraries were used: dplyr^49^, ggplot2^50^, Biostrings^51^, tidyr^52^. For Python, library Pandas^53^ was used.

### Binding assays

All binding assays were performed in a total reaction volume of 100 µL of binding buffer. Prior to incubation, the aptamer was folded (by heating at 95 °C for 5 min and subsequent incubation at 21 °C for 30 min) and the cells were washed twice in binding buffer. For testing the binding affinity (K_D_ determination), 10^7^ *E. faecalis* cells were incubated with various concentrations of aptamer (final aptamer concentrations: 0, 1, 3, 10, 30, 100, and 300 nM) for 30 min at 21 °C while shaking at 550 rpm. After incubation, unbound aptamers were removed by centrifugation for 10 min at 13,000×*g* and washing the cell pellet three times with 1 mL fresh binding buffer (centrifugation steps in between). Cell pellets were subsequently resuspended in 50 µL nuclease-free water. For specificity testing, the aptamer was incubated with ten different *Enterococcus* spp. (10^7^ cells each) and ten different non-*Enterococcus* spp. (10^7^ cells each) at a final aptamer concentration of 100 nM. The amount of cell-bound aptamer was finally measured via qPCR. Three biological replicates were performed for each tested aptamer concentration and bacterial species, respectively. The K_D_ (dissociation constant) was determined by a non-linear regression analysis using SigmaPlot v14.0 (Systat Software, Inc.)^54^.

### Epifluorescence microscopy

For fluorescence microscopy studies, 10^7^ bacterial cells were incubated with 100 nM of 5′-FAM-labelled aptamer (binding buffer for blank reactions) in 100 µL binding buffer for 30 min at 21 °C and 550 rpm shaking. After the binding reaction, the aptamer-cell complexes were washed with 1 mL binding buffer and resuspended in a final volume of 500 µL binding buffer and the suspension filtered onto a 0.2 µm black polycarbonate filter membrane (25 mm diameter; Merck Isopore, GTBP02500). For SYBR Gold staining the filters were placed in a petri dish on a drop (30 µL) of SYBR Gold (10,000 × concentrate in DMSO, diluted 400-fold in sterile deionized water) and kept in the dark for 15 min. After incubation, the filters were washed three times with a few drops of sterile 1 × PBS. All filters were dried in the dark at room temperature for approximately 30 min and then mounted onto a microscopic slide with one drop of anti-fading mounting solution (Citifluor). Another drop of mounting solution was directly placed on the filter before adding the cover slip. To remove excess liquid, the prepared slide was carefully pressed between two layers of absorbent paper. Slides were examined with immersion oil using a Nikon Eclipse Ni microscope at 1000 × magnification (Ex ~ 470, Em ~ 515) equipped with a Nikon DS-Qi2 camera.

## Supplementary information

Supplementary Information 1.

Supplementary Information 2.

## Data Availability

The Nextflow script and additional script files are located in the github repository https://github.com/hovercat/aptaflow/releases/tag/1.0. Raw FASTQ files were uploaded to the Sequence Read Archive (SRA) at NCBI and can be downloaded using the accession number PRJNA615076. Results of the analysis workflow are given in the Supplementary Material but can also be reproduced using the workflow on the raw NGS data.
