# Supplementary material for: DNA aptamers against bacterial cells can be efficiently selected by a SELEX process using state-of-the art qPCR and ultra-deep sequencing
Source: Sci Rep. 2020 Dec 1;10:20917. doi: 10.1038/s41598-020-77221-9 (PMC7708460; doi:10.1038/s41598-020-77221-9)
Supplement: Supplementary file 1 — Supplementary Information 1. [file 41598_2020_77221_MOESM1_ESM.pdf]

DNA aptamers against bacterial cells can be efficiently selected by a SELEX process using state-of-the art qPCR and ultra-deep sequencing

Claudia Kolm[1,2], Isabella Cervenka[1,2], Ulrich J. Aschl[1,2], Niklas Baumann[1,2], Stefan Jakwerth[2,3], Rudolf Krska[4,5], Robert L. Mach[6], Regina Sommer[2,3], Maria C. DeRosa[7], Alexander K.T. Kirschner[2,3,8], Andreas H. Farnleitner[2,8,9], Georg H. Reischer[1,2,9]*

[1] TU Wien, Institute of Chemical, Environmental & Bioscience Engineering, Molecular Diagnostics Group, Department IFA-Tulln, Tulln, Austria; [2] ICC Interuniversity Cooperation Centre Water & Health, Vienna, Austria (www.waterandhealth.at); [3] Medical University Vienna, Institute for Hygiene and Applied Immunology, Vienna, Austria; [4] University of Natural Resources and Life Sciences, Vienna (BOKU), Department IFA-Tulln, Institute of Bioanalytics and Agro-Metabolomics, Tulln, Austria; [5] Queen's University Belfast, School of Biological Sciences, Institute for Global Food Security, Northern Ireland, United Kingdom; [6] TU Wien, Institute of Chemical, Environmental & Bioscience Engineering, Research Division Biochemical Technology, Research Group Synthetic Biology and Molecular Biotechnology, Vienna, Austria; [7] Carleton University, Department of Chemistry, Ottawa, Canada; [8] Karl Landsteiner University of Health Sciences, Research Unit Water Quality and Health, Krems, Austria; [9] TU Wien, Institute of Chemical, Environmental & Bioscience Engineering, Research Area Biochemical Technology, Research Group of Environmental Microbiology and Molecular Diagnostics, Vienna, Austria

* To whom correspondence should be addressed. Tel: [+43158801166556]; Email: [georg.reischer@tuwien.ac.at].

CONTENT:

1. Real-time monitored test-PCR reaction
2. Amplification and standard melting curves
3. Predicted secondary structures of aptamer candidates
4. Enrichment trajectories of aptamers
5. Bacterial strains
6. Next-generation sequencing workflow

1. Real-time monitored test-PCR reaction

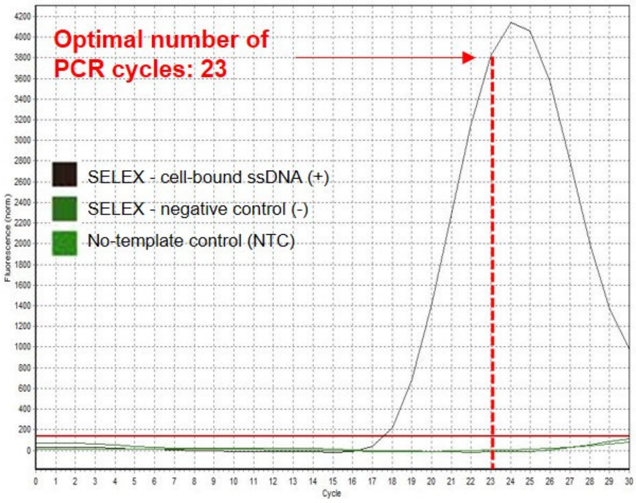

Supplementary Figure S1. Exemplary results from real-time monitored test-PCR reactions performed on an Eppendorf Realplex cycler with 1x EvaGreen fluorescence dye and 1 µL DNA template in total reaction volume of 25 µL. SELEX negative controls (cells only) and no-template controls (PCR-H_2O instead of DNA) were included in each run to check for contamination. The cycle number before the amplification curve reaches the fluorescence maximum (highest fluorescence signal) was used for subsequent preparative PCR amplification of cell-bound ssDNA fractions to ensure high amplification yield with no by-products.

2. Amplification and standard melting curves

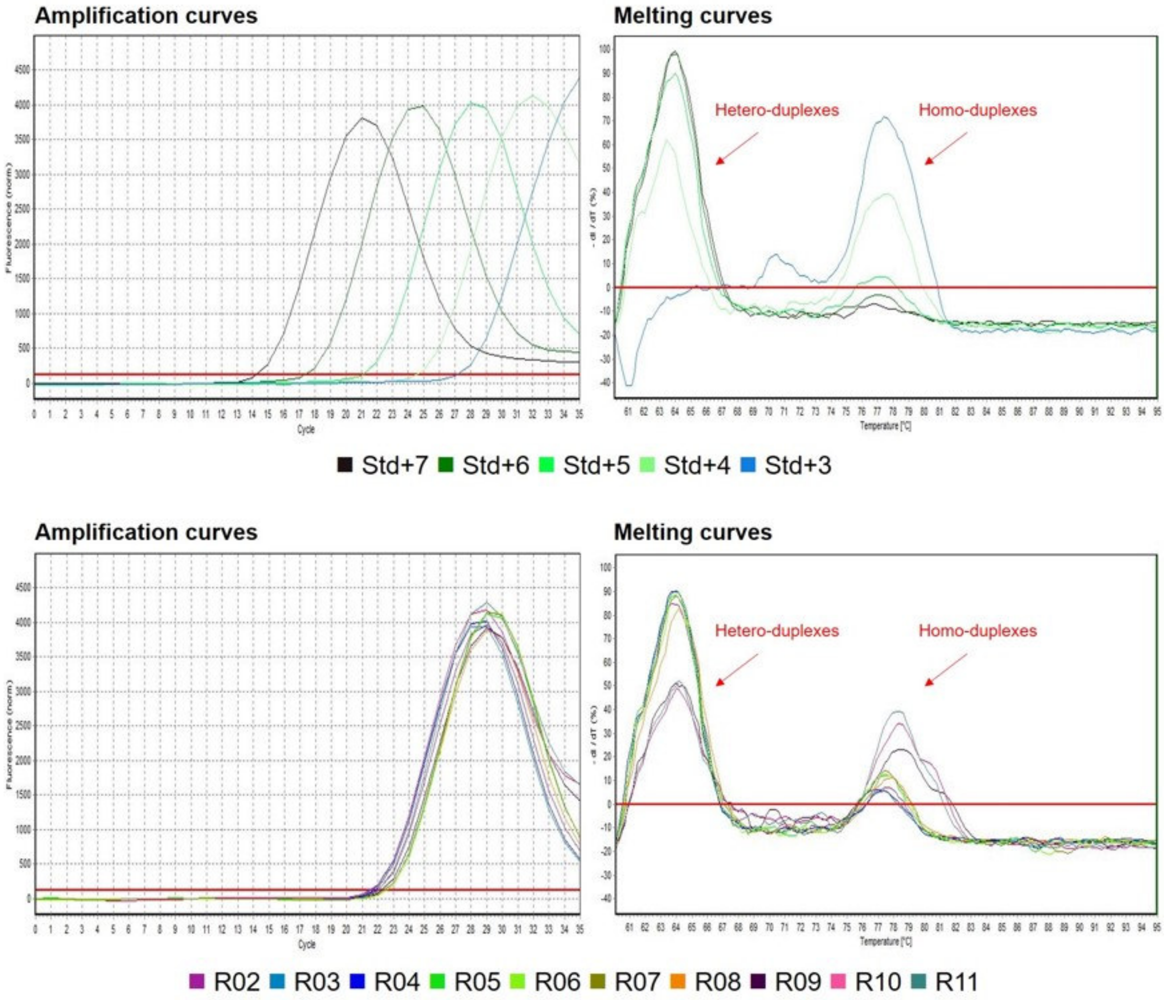

Supplementary Figure S2. Amplification and melting curves from random ssDNA library dilutions (Std. +7 to +3) used as reference and SELEX ssDNA pools (R02 to R11). As described in the Methods section (main manuscript), remelting curve analysis was performed after initial amplification and standard melting curve analysis. In contrast to remelting curves, standard melting curves were performed by total denaturation at 95 °C after amplification, short reannealing at 60 °C and then gradual heating to 95 °C. Reannealing at 60 °C resulted in the formation of hetero- and homo-duplexes (double peaks) that correlate with sequence diversity. Hetero-duplexes (T_m ~64 °C) are DNA products that match only partially due to common and complementary primer binding sites, while homo-duplexes (T_m ~78 °C) are stable complementary DNA products (the two complementary strands of a PCR product). As the diversity of the ssDNA pools to be analyzed decreases, the proportion of homo-duplexes formed increases. Vice versa, the higher the diversity, the more likely the formation of hetero-duplexes as the chances that complementary sequences find and hybridize to each other after total denaturation are low (see ssLib +7, upper right panel). Subsequent remelting curve analysis with reannealing at 70 °C was then performed to shift the detection window for better resolution by suppressing the reannealing of the primer binding sites and the formation of hetero-duplexes.

3. Predicted secondary structures of aptamer candidates

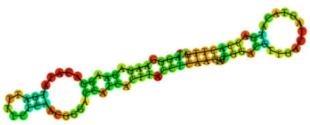

EF501 [MFE: -11.01 kcal/mol]

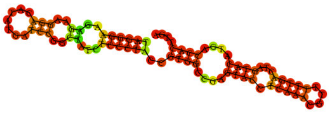

EF502 [MFE: -15.33 kcal/mol]

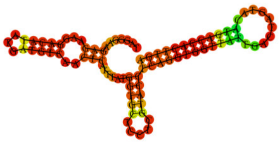

EF503 [MFE: -17.39 kcal/mol]

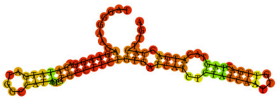

EF504 [MFE: -15.33 kcal/mol]

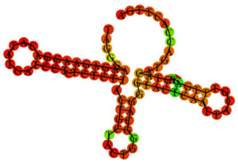

EF505 [MFE: -18.28 kcal/mol]

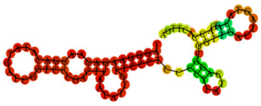

EF506 [MFE: -15.48 kcal/mol]

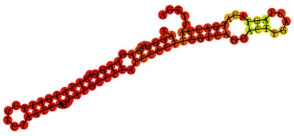

EF507 [MFE: -22.63 kcal/mol]

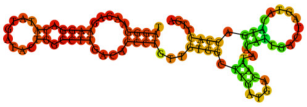

EF508 [MFE: -13.58 kcal/mol]

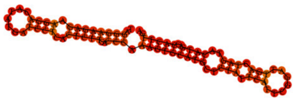

EF509 [MFE: -19.33 kcal/mol]

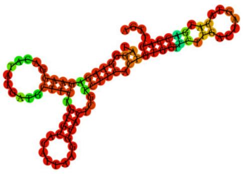

EF510 [MFE: -18.07 kcal/mol]

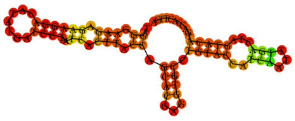

EF511 [MFE: -14.98 kcal/mol]

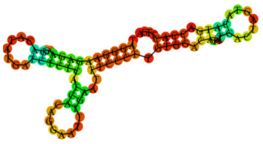

EF512 [MFE: -14.03 kcal/mol]

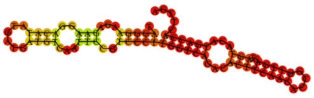

EF513 [MFE: -19.38 kcal/mol]

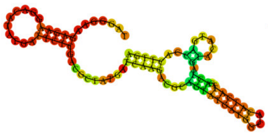

EF514 [MFE: -13.11 kcal/mol]

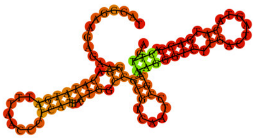

EF515 [MFE: -16.40 kcal/mol]

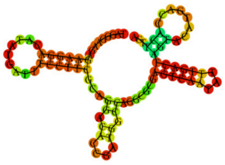

EF516 [MFE: -15.39 kcal/mol]

Supplementary Figure S3. Predicted secondary structures and minimal free energies (MFE) of aptamer candidates EF501-516 calculated by RNAfold.

4. Enrichment trajectories of aptamers

Supplementary Table S1. Enrichment trajectories of aptamers EF504, EF508 and EF513 over consecutive SELEX rounds based on NGS data analysis.

	SELEX Round	Absolute counts	Rank	Reads per million	% of pool population
**EF504**	R02	n.d.	n.d.	n.d.	n.d.
	R03	n.d.	n.d.	n.d.	n.d.
	R04	1	>#1000	6	0.003%
	R05	2	>#1000	12	0.007%
	R06	5	>#1000	25	0.012%
	R07	9	#192	53	0.031%
	R08	50	#5	245	0.120%
	R09	68	#9	425	0.266%
	R10	238	#7	1075	0.486%
	R11	322	#8	1370	0.583%
	**SELEX Round**	**Absolute counts**	**Rank**	**Reads per million**	**% of pool population**
**EF508**	R02	n.d.	n.d.	n.d.	n.d.
	R03	n.d.	n.d.	n.d.	n.d.
	R04	n.d.	n.d.	n.d.	n.d.
	R05	3	>#1000	18	0.011%
	R06	5	>#1000	25	0.012%
	R07	18	#9	105	0.061%
	R08	21	#70	103	0.050%
	R09	39	#50	244	0.153%
	R10	92	#60	416	0.188%
	R11	218	#21	928	0.394%
	**SELEX Round**	**Absolute counts**	**Rank**	**Reads per million**	**% of pool population**
**EF513**	R02	n.d.	n.d.	n.d.	n.d.
	R03	n.d.	n.d.	n.d.	n.d.
	R04	n.d.	n.d.	n.d.	n.d.
	R05	n.d.	n.d.	n.d.	n.d.
	R06	4	>#1000	20	0.011%
	R07	1	>#1000	6	0.004%
	R08	4	>#1000	20	0.010%
	R09	21	#233	131	0.082%
	R10	52	#183	235	0.106%
	R11	71	#216	302	0.129%

(n.d. = not determined)

5. Bacterial Strains

In this study, glycerol stocks of the bacteria were used for the *in vitro* selection and characterization of the aptamers. These stocks were prepared for all organisms according to the following procedure. A single bacterial colony from agar plates was transferred into a sterile 50 mL test tube containing 10 mL liquid broth (tryptic soy broth or brain-heart infusion broth) and incubated overnight at the corresponding temperature (37 or 30 °C). On the next day, 40 mL of fresh broth was inoculated with ~ 1 mL of the overnight culture and the cells were grown until mid-exponential growth phase (based on pre-monitored growth curves, Supplementary Figure S4 and S5). After cultivation, the cell suspension was gently mixed with sterile glycerol (final concentration: 20%). To ensure that the glycerol efficiently penetrates the cell, the cell suspension with added glycerol was kept at room temperature for at least 15 min. Subsequently cryogenic storage tubes were filled with 1 mL aliquots of the glycerol-cell suspension, placed in liquid nitrogen and immediately stored at -80 °C until further use.

Bacterial cell counts of the glycerol stocks were determined via flow cytometry using the Attune NxT Acoustic Focusing Cytometer (Life Technologies, Darmstadt, Germany) equipped with a 488 nm flat-top laser at 50 mW. Cells were analyzed after SYBR Green I staining according to the following procedure. Aliquots of each bacterial glycerol stock were thawed, transferred into 1.5 mL tubes and centrifuged at 13,000 x g for 10 min. The cell pellets were washed twice with 1 mL sterile 1x PBS, pH 7.4 by centrifugation at 13.000 x g for 10 min and resuspended in a total volume of 500 µL buffer. For cell counting, cells were diluted 1:100 or 1:1,000 in sterile 1x PBS to final reaction volume of 1 mL and stained with 10 µL SYBR Green I. Cultivation conditions and flow cytometry results are summarized in Supplementary Table S2.

Supplementary Table S2. Cultivation conditions of all bacterial species used in this study, including OD_{600} values during cell harvesting and flow cytometrically measured cell counts per mL. All strains were cultivated under aerobic conditions in either tryptic soy broth (TSB) or brain-heart infusion (BHI) broth while shaking at 150 rpm. Cell counts are given as the mean cell concentration of three biological replicates (SD: standard deviation of the mean)

Strain No.		Growth			OD_{600}	Cell concentration [mL^{-1}]	
		Medium	Temp.	Time		Mean	SD
DSM 20478	*Enterococcus faecalis*	TSB	37 °C	150 min	1.064	5.41E+08	7.04E+06
DSM 20477	*Enterococcus faecium*	TSB	37 °C	150 min	0.524	2.37E+08	1.32E+07
DSM 20633	*Enterococcus durans*	TSB	37 °C	150 min	0.720	7.71E+07	5.32E+06
DSM 20160	*Enterococcus hirae*	TSB	37 °C	135 min	0.924	4.95E+08	1.10E+07
DSM 20628	*Enterococcus gallinarum*	TSB	37 °C	240 min	1.684	8.71E+08	2.60E+07
DSM 20680	*Enterococcus casseliflavus*	TSB	37 °C	180 min	1.244	8.97E+08	9.01E+06
DSM 4838	*Enterococcus mundtii*	TSB	37 °C	150 min	1.370	6.96E+08	9.92E+06
DSM 6905	*Enterococcus sulfureus*	TSB	37 °C	180 min	2.920	3.32E+08	1.63E+07
DSM 20682	*Enterococcus cecorum*	TSB	37 °C	210 min	1.180	9.42E+08	7.71E+06
DSM 20679	*Enterococcus avium*	TSB	37 °C	210 min	0.782	4.54E+07	3.10E+06
DSM 11492	*Enterococcus asini*	TSB	37 °C	210 min	0.590	2.26E+08	8.88E+06
DSM 20480	*Streptococcus bovis*	TSB	37 °C	150 min	1.195	5.44E+08	5.73E+06
DSM 20560	*Streptococcus salivarius*	TSB	37 °C	240 min	0.890	1.13E+08	3.20E+07
DSM 20481	*Lactococcus lactis*	BHI	37 °C	240 min	0.530	3.36E+07	2.32E+06
DSM 20232	*Staphylococcus aureus*	BHI	37 °C	120 min	0.855	1.80E+08	9.86E+06
NCTC 9001	*Escherichia coli*	BHI	37 °C	120 min	1.705	1.91E+08	9.45E+07
ATCC 25922	*Escherichia coli*	BHI	37 °C	150 min	2.275	8.35E+08	9.29E+07
ATCC 8739	*Escherichia coli*	BHI	37 °C	120 min	0.892	6.98E+08	1.14E+08
DSM 30039	*Citrobacter freundii*	BHI	30 °C	180 min	1.130	2.25E+08	1.03E+07
NCTC 9528	*Klebsiella aerogenes*	BHI	30 °C	210 min	1.050	1.92E+08	1.42E+07
NCTC 10662	*Pseudomonas aeruginosa*	BHI	37 °C	180 min	1.235	4.40E+08	6.50E+06

TSB: 30 g of Caso Broth (article no. X938, Roth, Germany) and 3 g of yeast extract (article no. 2904, Roth, Germany) for 1000 mL broth
BHI: 37 g of Brain Heart Infusion (article no. X916, Roth, Germany) for 1000 mL broth

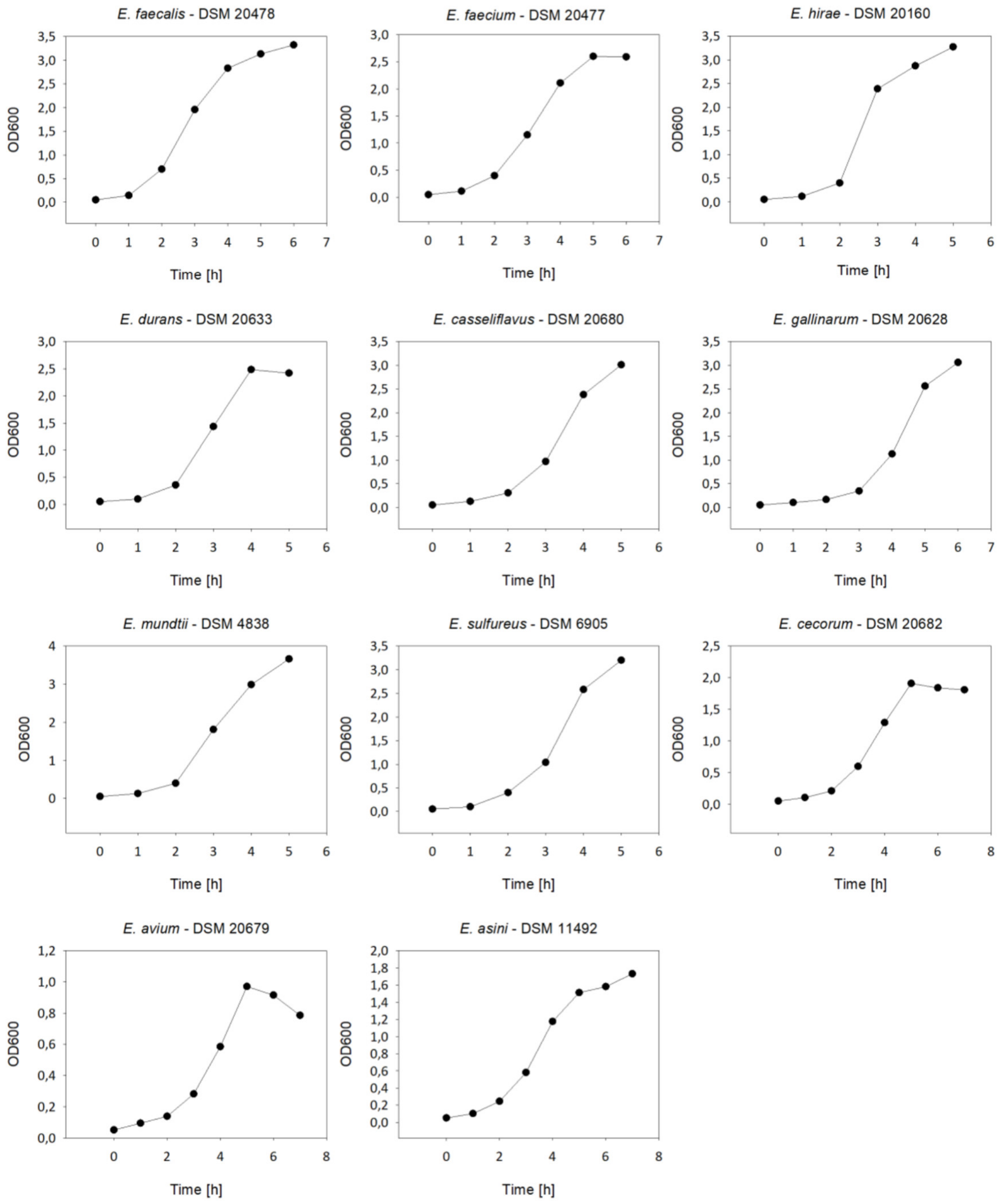

Supplementary Figure S4. Growth curves of all *Enterococcus* spp. used in this study (n = 11). Cells were grown in tryptic soy broth and monitored over a time period of five to seven hours to determine the exponential growth phase for subsequent glycerol stock preparation. OD_{600} measurements were carried out every hour.

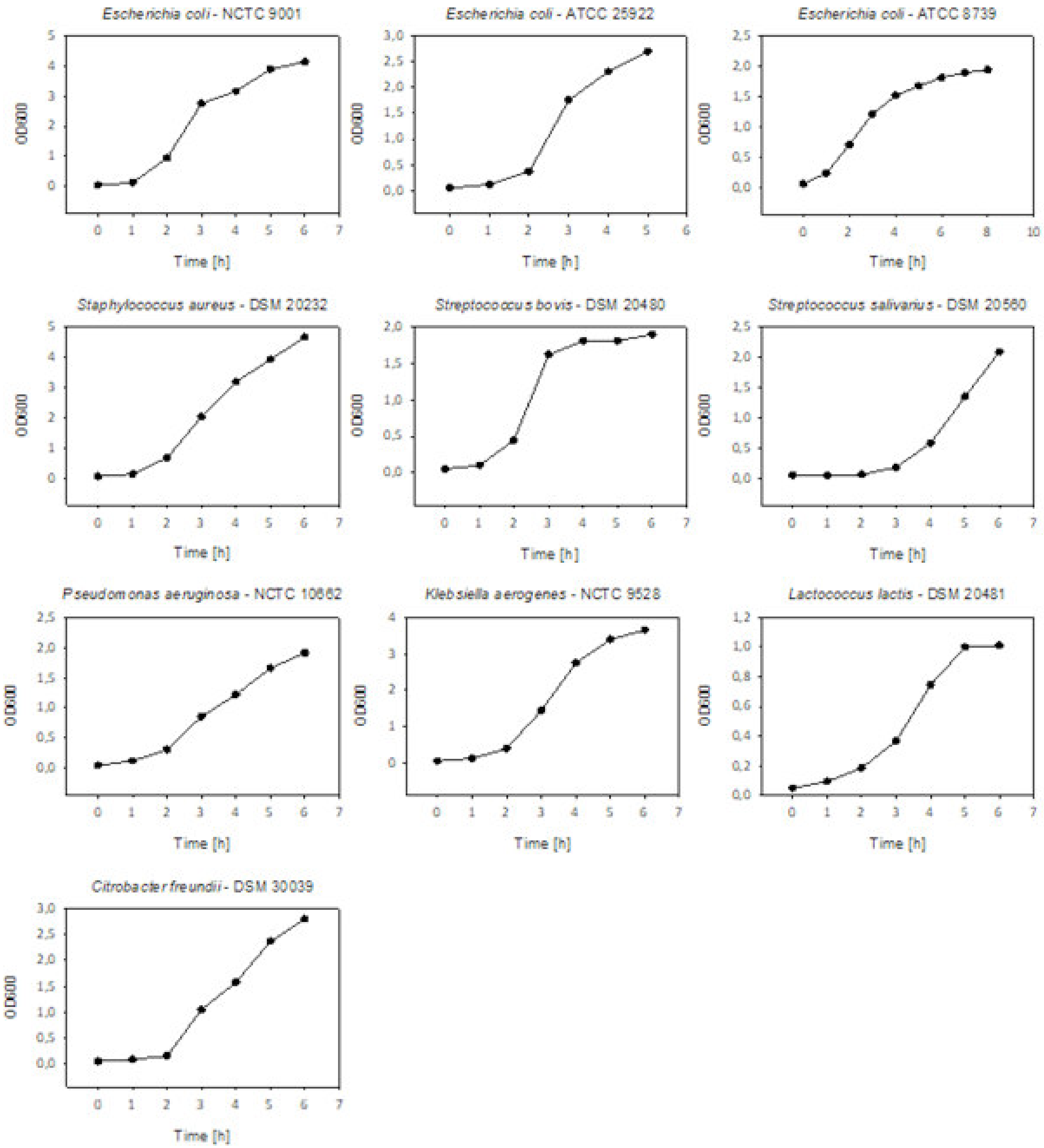

Supplementary Figure S5. Growth curves of all non-*Enterococcus* spp. used in this study (n = 10). Cells were grown in either tryptic soy broth or brain-heart infusion broth and monitored over a time period of five to seven hours to determine the exponential growth phase for subsequent glycerol stock preparation. OD_{600} measurements were carried out every hour.

6. Next-generation sequencing and bioinformatical workflow

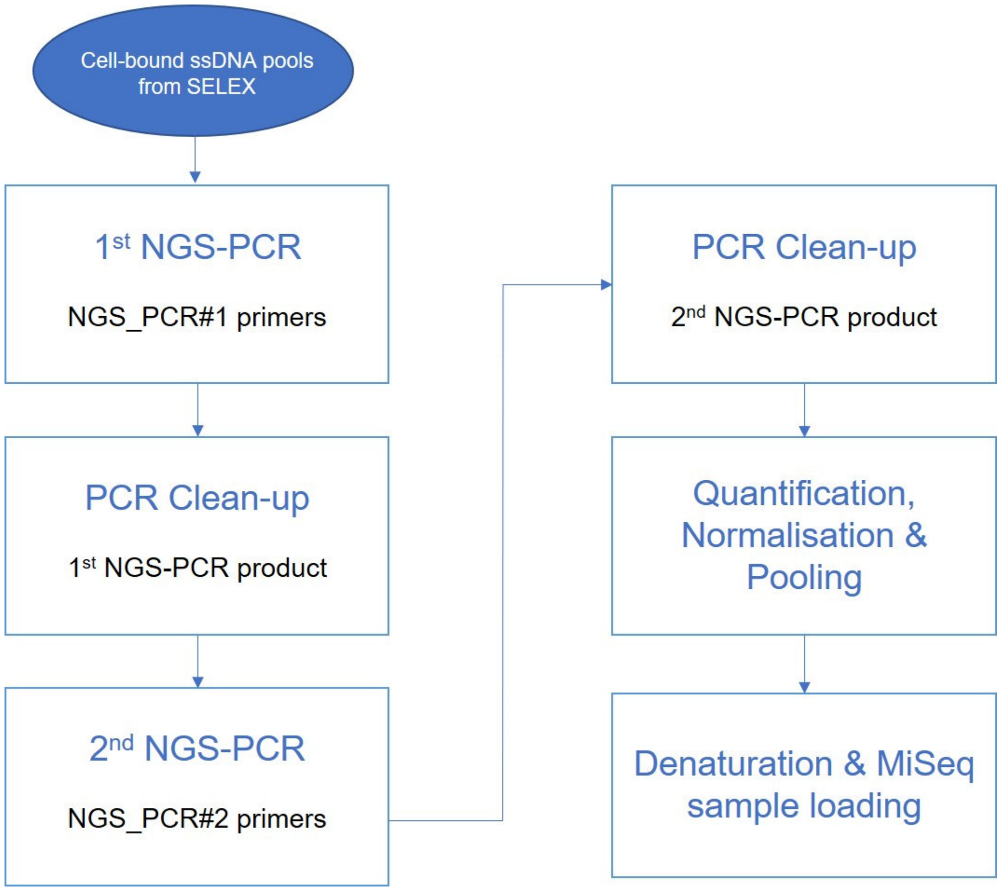

Supplementary Figure S6. NGS sample preparation workflow used in this study

Supplementary Table S3. Primer sequences used for NGS-library preparation of SELEX pools. Primer regions highlighted in blue hybridize at the constant primer binding regions of the ssDNA SELEX pools (SELEX-specific sequence). Sequencing primer regions are highlighted in red, the overhang regions in green and the flow cell adapter regions in purple. The overhang regions of NGS_PCR#1 primers serve as priming sites for NGS-PCR#2 primers.

Name	Sequence (5'-3')
NGS_PCR#1_forward	TCGTCGGCAGCGTCAGATGTGTATAAGAGACAGTAGGGAAGAGAAGGACATATGAT
NGS_PCR#1_reverse	GTCTCGTGGGCTCGGAGATGTGTATAAGAGACAGTCAAGTGGTCATGTACTAGTCAA
NGS_PCR#2_forward	CAAGCAGAAGACGGCATACGAGAT - [i7 index] - GTCTCGTGGGCTCGG
NGS_PCR#2_reverse	AATGATACGGCGACCACCGAGATCTACA - [i5 index] – TCGTCGGCAGCGTC

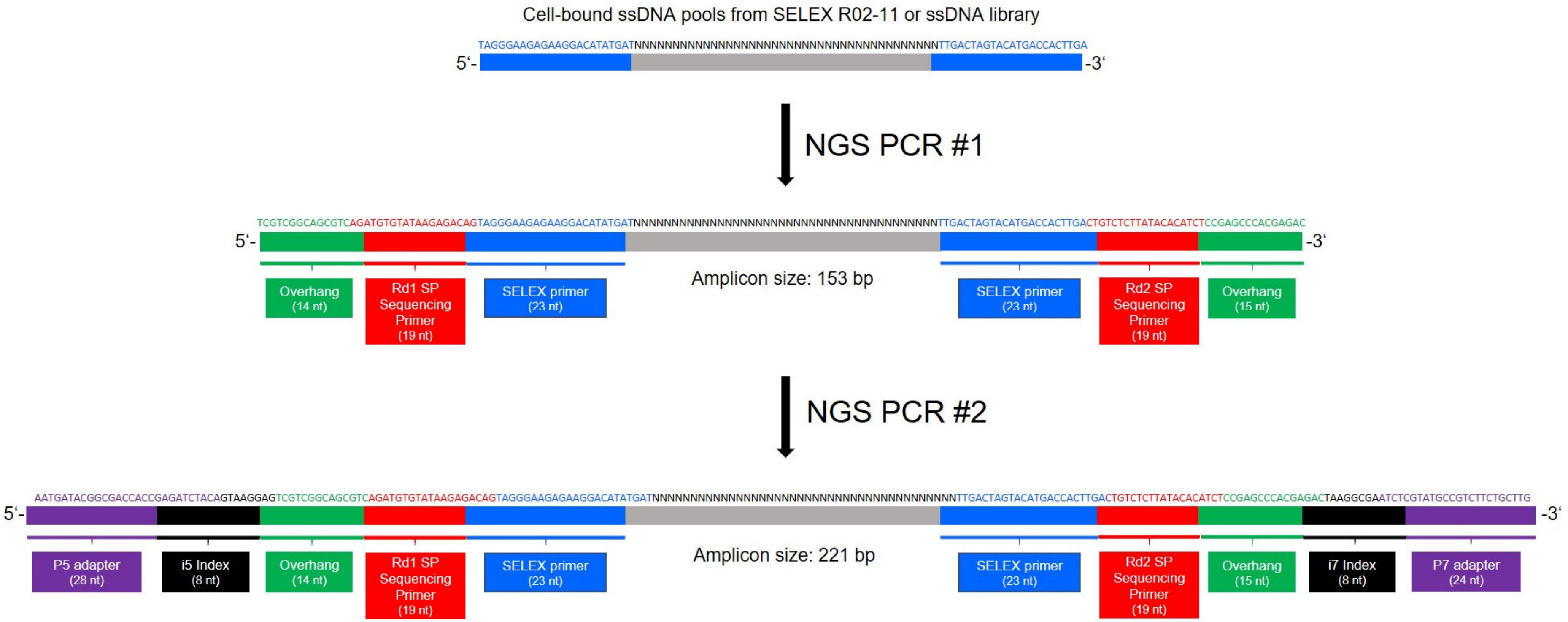

Supplementary Figure S7. NGS amplicon workflow. A two-step PCR procedure was used to attach sequencing primer, barcodes (i5 and i7 index) and flow cell adapters.

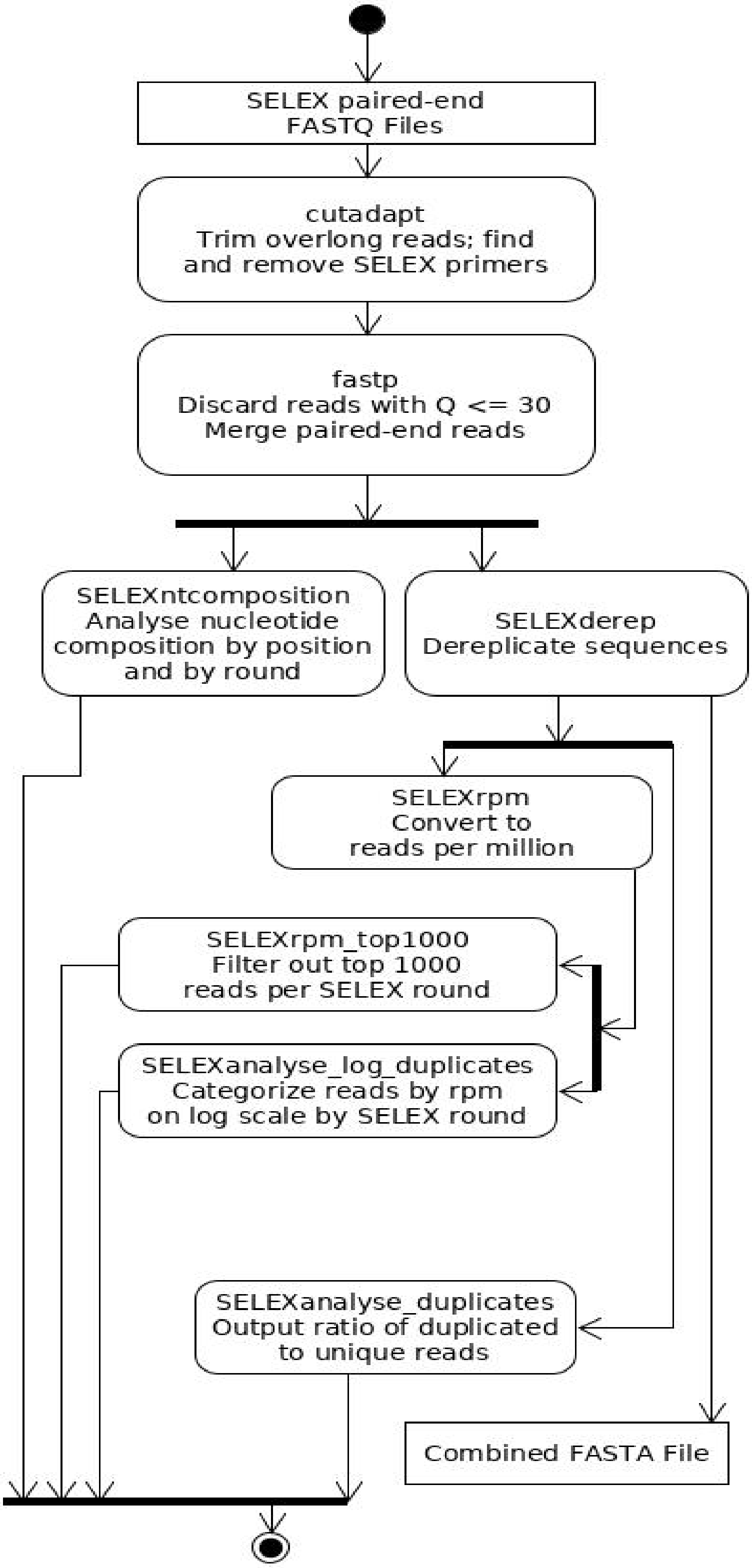

Supplementary Figure S8. Schematic illustration of the NGS data analysis workflow used in this study.
